# Single-cell RNA sequencing unveils an IL-10-producing helper subset that sustains humoral immunity during persistent infection

**DOI:** 10.1038/s41467-018-07492-4

**Published:** 2018-11-28

**Authors:** Gang Xin, Ryan Zander, David M. Schauder, Yao Chen, Jason S. Weinstein, William R. Drobyski, Vera Tarakanova, Joseph Craft, Weiguo Cui

**Affiliations:** 1 Blood Research Institute, BloodCenter of Wisconsin, Milwaukee, WI 53213 USA; 2 Department of Microbiology and Molecular Genetics, Medical College of Wisconsin, Milwaukee, WI 53226 USA; 3 Department of Immunobiology, Yale University School of Medicine, New Haven, CT 06520 USA

## Abstract

During chronic viral infection, the inflammatory function of CD4 T-cells becomes gradually attenuated. Concurrently, Th1 cells progressively acquire the capacity to secrete the cytokine IL-10, a potent suppressor of antiviral T cell responses. To determine the transcriptional changes that underlie this adaption process, we applied a single-cell RNA-sequencing approach and assessed the heterogeneity of IL-10-expressing CD4 T-cells during chronic infection. Here we show an IL-10-producing population with a robust Tfh-signature. Using IL-10 and IL-21 double-reporter mice, we further demonstrate that IL-10^+^IL-21^+^co-producing Tfh cells arise predominantly during chronic but not acute LCMV infection. Importantly, depletion of IL-10^+^IL-21^+^co-producing CD4 T-cells or deletion of *Il10* specifically in Tfh cells results in impaired humoral immunity and viral control. Mechanistically, B cell-intrinsic IL-10 signaling is required for sustaining germinal center reactions. Thus, our findings elucidate a critical role for Tfh-derived IL-10 in promoting humoral immunity during persistent viral infection.

## Abstract (web summary)

During chronic infection CD4+ T cells can progressively acquire IL-10 producing functionality. Here the authors use single cell RNA sequencing to interrogate the IL10 CD4+ T cell compartment in a murine model of chronic infection and identify Il10-producing Tfh involved in promotion of the antiviral humoral immune response.

## Introduction

CD4 T cells display immense versatility in changing their differentiation pattern in the face of persistent lymphocytic choriomeningitis virus (LCMV) infection^1^. Similar to CD8 T cells, CD4 T cells rapidly lose their capacity to produce the effector cytokines IL-2, TNF-α, and IFN-γ during chronic infection^2,3^. However, CD4 T cells also gradually acquire the capacity to express IL-21 and IL-10^4,5^, suggesting that continuous antigenic exposure may drive functional adaption within the T helper cell compartment. Notably the inhibitory role of IL-10 in suppressing T-cell responses during chronic viral infection is well-documented^6–8^. However, IL-10 signaling may also protect the host against collateral damage caused by excessive and prolonged inflammation^9^. Intriguingly, two recent studies have identified that the regulatory effects of IL-10 may be multifaceted, and can largely depend on the cellular source of IL-10, the responding cell type, and the nature of the infection^4,10^. Although multiple distinct CD4 T-cell subsets, including Tregs, Tr1 cells, and Th1 cells can produce IL-10 in response to viral infection^4,10,11^, the biological consequences of IL-10 derived from T helper cell subsets other than that of Th1 cells remains incompletely understood in the context of persistent infection.

In contrast to the suppressive nature of IL-10, CD4 T-cell-derived IL-21 is critical to sustain the function of CD8 T-cells and mediate viral containment during persistent infection^5,12–14^. IL-21 is also a potent facilitator of B cell help^15^. Recent evidence suggests that CD4 T follicular helper (Tfh) cells are the major producers of IL-21 during chronic viral infection^1^. Several studies over the last decade have identified that Tfh cells play a central role in orchestrating the germinal center (GC) reaction, a process that is essential for the selection of high-affinity B cell receptors and the development of long-lived plasma cells and memory B cells^16–20^. Despite the pivotal role of Tfh cells in mediating humoral immunity during chronic infections, the cellular and molecular factors important for Tfh differentiation and function are still being unraveled.

Tfh cells can be distinguished from other CD4 T-cell lineages based on their combinatorial expression of the chemokine receptor CXCR5, the co-stimulatory receptor ICOS, and the transcriptional repressor B cell lymphoma 6 (Bcl-6), all of which are required for Tfh differentiation^21,22^. Additionally, CD4 T-cell expression of SLAM-associated protein (SAP) is essential for facilitating the formation of stable T-cell–B-cell conjugates and is critical for GC Tfh development^16,23,24^. Although the importance of Tfh-secreted IL-21 in maintaining the GC reaction is well-appreciated, several recent reports have identified that Tfh cells display vast heterogeneity in the effector molecules they produce^25–29^. However, the importance of Tfh-derived cytokines other than IL-21 remains less well-defined. In this study, we performed single-cell RNA sequencing (scRNA-seq) to determine the heterogeneity among IL-10-secreting CD4 T cells during persistent viral infection. Unexpectedly, single-cell transcriptomics uncovered a subset of IL-10-producing CD4 T cells with a robust Tfh signature. Herein, we report that a unique subset of IL-10^+^IL-21^+^Tfh cells predominantly arise during chronic, but not acute LCMV infection. Importantly, depletion of IL-10^+^IL-21^+^ co-producing CD4 T cells or Tfh-specific deletion of IL-10 results in significantly reduced GC reactions, antibody production, and viral control. Collectively, this study highlights the importance of Tfh cells remaining plastic in their ability to produce cytokines so that they can optimally regulate humoral immune responses to establish control over viral replication.

## Results

### scRNA-seq reveals a subset of IL-10^+^Tfh cells

**Fig. 1:**
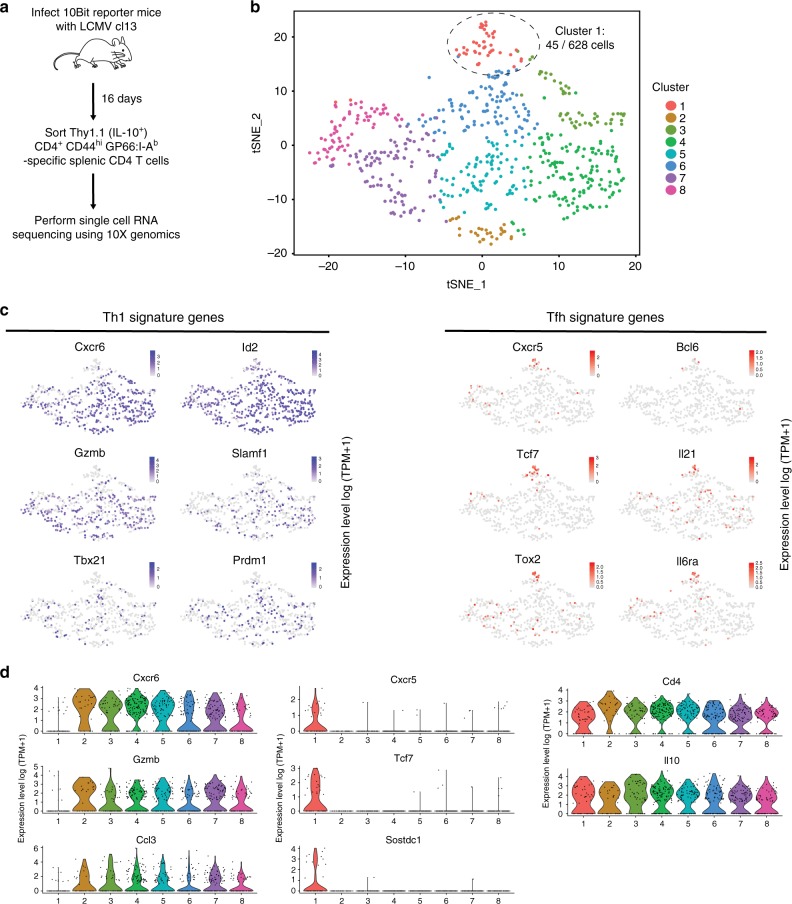
ScRNA-seq of LCMV-specific IL-10^+^CD4 T cells. **a** Outline of experiment. **b** *t*-SNE projection of 628 IL-10-producing CD4 T cells from LCMV Cl13-infected mice at day 16 p.i. colored by cluster. **c** *t*-SNE plots showing relative expression of Th1 (left) and Tfh (right) signature genes in each cell. **d** Violin plots depicting expression of control genes (*Cd4*, *Il10*), Th1 genes (*Ccl3*, *Cxcr6*, *Gzmb*), and Tfh genes (*Cxcr5*, *Tcf7*, *Sostdc1*). See also Figure S1

To investigate the transcriptional heterogeneity of IL-10-producing CD4 T cells responding to chronic viral infection, we performed scRNA-seq on IL-10-expressing CD4 T cells from mice infected with persistent LCMV Cl13. To do this, 10BiT reporter mice (which possess a BAC transgene containing the *Il10* gene locus with the Thy1.1 cDNA insertion^30^) were infected with LCMV Cl13, and on day 16 post infection (p.i.), we sort-purified Thy1.1^+^(IL-10^+^)CD4 T cells specific for the I-A^b^ restricted GP_61-80_ epitope of LCMV (Fig. 1a). Next, we used the 10X Genomics Chromium system to generate a scRNA-seq library (Fig. 1a), and a downstream transcriptional analysis was performed on 628 IL-10-expressing CD4 T cells.

Notably, IL-10^+^CD4 T cells grouped distinctly into eight clusters when visualized by *t*-distributed stochastic neighbor-embedding analysis (*t*-SNE) projection (Fig. 1b). As expected, many IL-10-expressing CD4 T cells co-expressed the gene *Tbx21*, which encodes the Th1-defining transcription factor T-bet (Fig. 1c). Moreover, genes encoding Th1-associated molecules granzyme B, Ccl3, Cxcr6, and Id2 were also highly expressed in most of these subpopulations (Fig. 1c, d and Supplementary Figure 1A). In particular, clusters 2–8, coordinately displayed high expression of these Th1-signature genes (Fig. 1c, d), suggesting that these subpopulations may be of the Th1 lineage. Additionally, the Blimp-1-encoding gene *Prdm1*, which has recently been identified as being essential for T-cell production of IL-10^4,31^, was ubiquitously expressed and was detected among all eight IL-10^+^T-cell clusters (Fig. 1c). Similarly, each cluster displayed high expression levels of *Maf* (encodes c-Maf), a potent inducer of IL-10 expression^32,33^ (Supplementary Figure 1B). Intriguingly, our scRNA-seq analysis also identified a unique cluster of IL-10-expressing CD4 T cells that displayed high expression of Tfh signature genes, including *Cxcr5*, *Il21*, *Tcf7*, and *Il6ra* (Fig. 1c, d). Moreover, *Bcl6* expression was also enriched within this Tfh-like subset (Fig. 1c). Of note, all eight clusters of IL-10^+^CD4 T cells displayed minimal expression of Foxp3 (Supplementary Figure 1C), suggesting the absence of the Treg lineage among GP_61-80_-specific IL-10-producing CD4 T cells. Lastly, among the Th1-like clusters 2–8, clusters 7 and 8 displayed relatively higher expression levels of *Gzmk*, *Klf2*, *Lgals1*, and *Lgals3*, indicating that these two subgroups may be at a different stage of differentiation compared to their Th1-like effector counterparts (Supplementary Figure 1D). Collectively, our scRNA-seq analyses highlight that IL-10-producing CD4 T cells responding to chronic viral infection are comprised of multiple transcriptionally distinct subsets.

### IL-10^+^IL-21^+^Tfh cells form during chronic but not acute LCMV

**Fig. 2:**
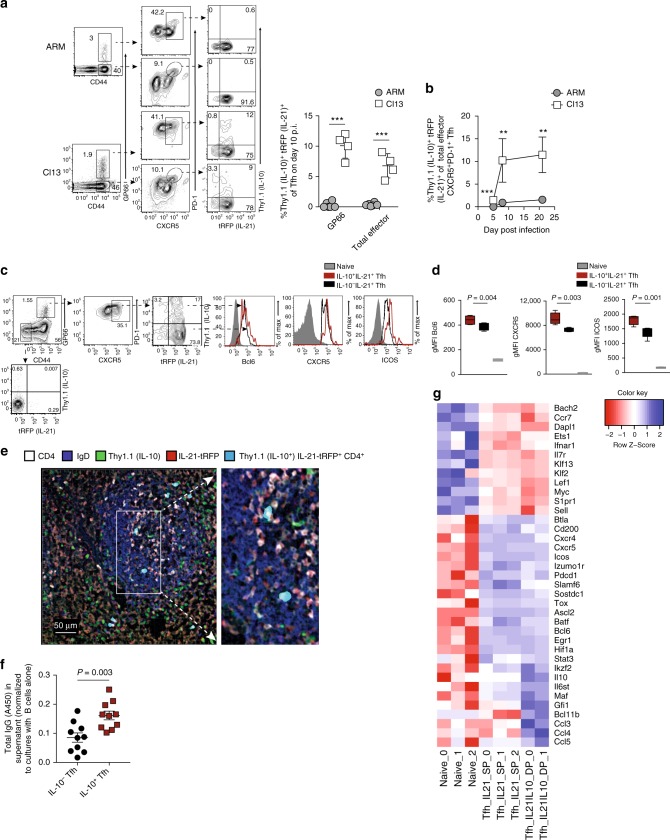
IL-10^+^IL-21^+^ Tfh cells largely develop during chronic but not acute LCMV. **a**, **b** *10BiT-Il21-RFP* mice were infected with either LCMV Arm or LCMV Cl13, and splenic CD44^+^ CD4 T cells were examined on day 10 p.i. **a** Representative flow plots and summary data displaying the frequency of CXCR5^+^ PD-1^hi^ effector and GP66:I-A^b^–specific Tfh cells and subsequent expression of IL-10(Thy1.1) and IL-21(tRFP). **b** Summary kinetics showing the proportion of IL-10^+^IL-21^+^ Tfh cells during LCMV Cl13 and ARM infection. **c**–**g** *10BiT-Il21-RFP* mice were infected with LCMV Cl13. **c**, **d** Flow plots (**c**) and summary data (**d**) depicting the relative expression of Bcl-6, CXCR5 and ICOS on IL-10^+^ IL-21^+^ and IL-10^-^IL-21^+^GP-66^+^Tfh cells on day 14 p.i. Gray histograms show the relative expression of these molecules in naive (CD44^lo^) CD4 T cells. **e** Microscopy of GCs from *10BiT-Il21-RFP* mice on day 21 p.i.; stained for IgD (blue) CD4 (white) Thy1.1 (green), and IL-21(red). White arrowheads point to Thy1.1^+^ (IL-10)IL-21^+^CD4 T cells found in B cell follicle. **f** Thy1.1^+^(IL-10)^+^ and Thy1.1^−^(IL-10^-^) Tfh cells were sort-purified from LCMV Cl13-infected *10BiT-Il21-RFP* mice on day 14 p.i. and co-cultured with Naive (IgM^+^ B220^+^) B cells in the presence of α nti-CD3 and αnti-IgM antibodies as previously described^36^. Summary graph (**f**) showing the relative amount of total IgG secreted in the supernatant after 5 days of culture. **g** Heatmap generated from bulk RNA-sequencing analysis depicting relative expression of select genes in IL-21^+^ and IL-10^+^IL-21^+^Tfh cells as compared to naïve CD4 T cells. IL-21^+^ and IL-10^+^IL-21^+^ Tfh cells (CD44^hi^ CXCR5^+^ PD-1^hi^) were sorted from the spleens of LCMV Cl13-infected mice on day 18 p.i. FPKM values were log-transformed and then centered and scaled for each row. Summary data (mean + /- SD in **a** and **b**, or box and whiskers plot showing median (midline) and interquartile range with upper and lower whiskers representing the range of data distribution in **d**) are from 4 to 6 mice/group and are representative of 2–3 independent experiments. Summary data (mean + /- SEM in **f**) are pooled data from three independent experiments. *P < 0.05, **P < 0.01, ***P < 0.0001, (N.S. = not significant). Data were analyzed using two-tailed unpaired student’s *t* tests. See also Figure S2

Our finding that a subset of IL-10-expressing CD4 T cells displays a robust Tfh signature was highly unexpected, and suggests that Tfh cells may also display remarkable heterogeneity. It also brings to question whether the development of IL-10^+^ Tfh cells is a distinct feature of chronic viral infections. Thus we next performed comparative studies to determine whether acute or chronic viral infection differentially supports the formation of IL-10^+^IL-21^+^ co-producing Tfh cells. To do this, we crossed 10BiT mice with IL-21-turbo red fluorescent protein (IL-21tRFP) reporter mice^12,25,34^ to generate 10BiT*-Il21*tRFP double-reporter mice. We then infected separate groups of 10BiT*-Il21*tRFP mice with LCMV Armstrong (Arm) or LCMV Cl13 to establish an acute or chronic viral infection, respectively. Of note, and in align with previous reports^4,25,34^, we found that Thy1.1 and IL-21-tRFP expression closely correlated with IL-10 and IL-21 protein production, respectively in virus-specific CD4 T cells (Supplementary Figure 2A, B). On day 10 p.i., the proportion of CXCR5^+^PD-1^hi^Tfh cells specific for LCMV GP_61-80_ epitope was similar between LCMV-Arm and LCMV Cl13-infected mice (Fig. 2a). Consistent with prior studies^1,35^, splenic Tfh cells from either acute or chronic LCMV-infected mice were highly competent to produce IL-21, with 75–90% of GP66:I-A^b^-specific Tfh cells actively producing IL-21 directly ex vivo (Fig. 2a). Strikingly, whereas Tfh cells responding to acute LCMV-Arm displayed a negligible capacity to co-produce both IL-10 and IL-21, ~10–15% of virus-specific and total effector Tfh cells from LCMV Cl13-infected mice were competent to co-produce IL-10 and IL-21 (Fig. 2a). This pattern of IL-10^+^IL-21^+^ dual-producing Tfh cells emerging primarily during chronic LCMV infection was temporally sustained (Fig. 2b, Supplementary Figure 2C–E).

To determine whether a more prolonged acute viral infection can result in the induction of IL-10^+^IL-21^+^Tfh cells, we infected 10BiT*-Il21*tRFP reporter mice with influenza A/PR8/34 (PR8), which is eliminated from the host within 10 days. Intriguingly, a minor population of Tfh cells responding to PR8 infection also co-produced IL-10 and IL-21 (Supplementary Figure 2F), suggesting that certain acute infections may also spur the development of IL-10^+^IL-21^+^Tfh cells. However, in contrast to chronic LCMV infection, IL-10^+^IL-21^+^Tfh cells rapidly declined in influenza-infected hosts (Supplementary Figure 2F). Taken together, these results demonstrate that Tfh cells responding to chronic viral infection exhibit an enhanced and sustained capacity to coordinately produce both IL-10 and IL-21.

We next investigated whether additional phenotypic and functional differences exist between IL-10^+^IL-21^+^ and IL-10^-^IL-21^+^Tfh cells during LCMV Cl13 infection. An assessment of Tfh-associated molecules revealed that the relative expression of Bcl-6 was increased by ~25% in IL-10^+^IL-21^+^GP66^+^Tfh cells compared with IL-10^−^IL-21^+^Tfh cells (Fig. 2c, d). As Bcl-6 is critical for coordinating Tfh differentiation and function^16^, these data suggest that IL-10^+^IL-21^+^Tfh cells may be more GC-like in nature as compared to their IL-10^-^IL-21^+^Tfh counterparts. In alignment with this idea, GP66:I-A^b^-specific IL-10^+^IL-21^+^Tfh cells also displayed 25–40% increases in their relative expression of ICOS and CXCR5 (Fig. 2c, d), indicating that IL-10^+^IL-21^+^ dual-producing Tfh cells may have enhanced trafficking to or retention within the B-cell follicle. Correspondingly, IL-10^+^IL-21^+^CD4 T cells were found to localize in close proximity to B-cell follicles and could readily be detected within the GC (Fig. 2e and Supplementary Figure 2G). Lastly, we identified that naive IgM^+^B220^+^ B cells class-switched and secreted higher amounts of IgG (Fig. 2f) when co-cultured with sort-purified IL-10^+^Tfh cells as compared with IL-10^-^Tfh cells, suggesting that Tfh-derived IL-10 may promote B cell-secreted antibody responses in vitro.

To determine whether IL-10^+^IL-21^+^Tfh cells are transcriptionally distinct from IL-21^+^ single-producing Tfh cells, we sorted these respective populations from LCMV Cl13-infected reporter mice on day 18 p.i. and performed RNA-seq. As expected, *Il10* expression levels were significantly elevated in IL-10^+^IL-21^+^Tfh cells as compared with their IL-21-single-producing counterparts (Fig. 2g). Intriguingly, RNA-seq analyses also indicated that expression of *Maf* was increased >twofold in IL-10^+^Tfh cells (Fig. 2g). In addition, several genes encoding various transcription factors, chemokines, and cell surface ligands and receptors were differentially expressed between these two subsets (Fig. 2g). Thus, IL-10^+^IL-21^+^Tfh cells and IL-21^+^ single-producing Tfh cells are transcriptionally distinct T helper cell populations.

Of note, we did not detect Foxp3 expression at levels above background in either IL-10^+^ or IL-10^-^GP-66-specific Tfh cells (Supplementary Figure 3A). However, ~10–15% of total IL-10^+^CXCR5^+^PD-1^hi^CD44^hi^ CD4 T cells stably expressed Foxp3 during LCMV Cl13 infection (Supplementary Figure 3A, C). A similar but slightly reduced proportion of Foxp3-expressing CXCR5^+^PD-1^hi^CD44^hi^ CD4 T cells was also observed in LCMV Cl13-infected Foxp3-YFP reporter mice (Supplementary Figure 3B). Additionally, and consistent with previous reports^36,37^, we identified that Foxp3^+^CXCR5^+^PD-1^hi^ CD4 T cells displayed increased expression levels of CTLA4, CD25, and GITR, but lower levels of IL-21, as compared with Foxp3^−^Tfh cells (Supplementary Figure 3C). Taken together, these data suggest that the majority of IL-10^+^IL-21^+^CXCR5^+^PD-1^hi^ CD4 T cells that form during persistent viral infection are of the Tfh lineage, and are therefore distinct from Foxp3-expressing T follicular regulatory cells (TFRs).

### IL-10^+^IL-21^+^CD4 T cells are essential to sustain GC reaction

**Fig. 3:**
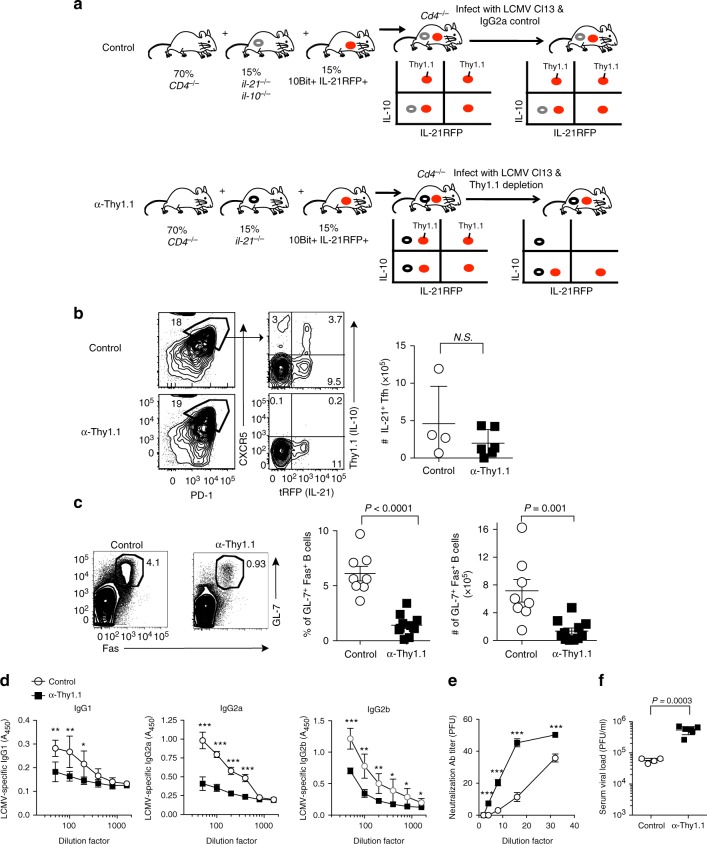
IL-10^+^IL-21^+^CD4 T cells promote humoral immunity during chronic LCMV. **a** Experimental scheme showing the generation of MBM chimeric mice and the strategy to delete IL-10^+^IL-21^+^ co-producing CD4 T cells. Briefly, CD4^*−/−*^ mice were lethally irradiated and reconstituted with bone marrow cells from the indicated donors at the ratios indicated. Red dots in the schematic represent bone marrow cells derived from*10BiT-Il21-RFP* donor mice. Gray dots and black dots represent bone marrow derived from il-21^*−/−*^IL-10^*−/−*^ and il-21^*−/−*^ donor mice respectively. The tables (right) indicate all of the potential IL-10 and IL-21-producing CD4 T cell compartments that bone marrow derived from the indicated donor mice can give rise to. **b**-**f** MBM mice were infected with LCMV Cl13 and treated with either Thy1.1 depletion or isotype control antibodies on days 4 and 6 p.i. **b**, **c** Representative flow plots and summary data showing the frequency and total number of Il-21^+^CXCR5^+^ PD-1^hi^ Tfh cells (**b**) and GL-7^+^Fas^+^ GC B cells (**c**) on day 21 p.i. (**d**) Summary graphs of LCMV-specific serum IgG1, IgG2a, and IgG2b antibodies. **e** Summary graph showing relative antibody neutralization titers for control and Thy1.1-depleted mice. **f** Viral titers were quantified by plaque assay using serum from experimental mice. Summary data (mean + /- SD in **b**) or (mean + /- SEM in **c**-**f**) are from 4 to 6 mice/group per experiment and are representative of two independent experiments. *P < 0.05, **P < 0.01, ***P < 0.0001, (N.S. = not significant). Data were analyzed using two-tailed unpaired student’s *t* tests. See also Figure S4

Considering the importance of B cell-secreted antibody responses in controlling persistent infection^1^, we hypothesized that coordinate production of IL-10 and IL-21 by CD4 T cells may be essential for sustaining virus-specific antibody responses. To test this, we took advantage of Thy1.1 expression on the surface of IL-10-producing cells in the 10BiT model to selectively deplete IL-10^+^IL-21^+^ co-producing CD4 T cells in mixed bone marrow (MBM) chimera experiments. To do this, we reconstituted lethally irradiated *Cd4*^*−/−*^ mice with MBM cells from the following donors: *Cd4*^*−/−*^ (70%) + *Il21*^*−/−*^ (15%) + 10BiT*-Il21*tRFP (15%) as shown in Fig. 3a. In doing so, CD4 T cells in MBM chimeric mice will only be derived from *Il21*^*−/−*^ and 10BiT*-Il21*tRFP in a 50:50 ratio, while the rest of the immune system remains largely wild type (WT, 70% from *Cd4*^*−/−*^). This approach allows for selective depletion of IL-10^+^IL-21^+^CD4 T cells by administering anti-Thy1.1 depletion antibodies, while 50% of both IL-10 and IL-21 single-producing CD4 T cells will remain intact (Fig. 3a). To generate control MBM chimera mice that would contain approximately equivalent numbers of IL-10 and IL-21 single-producing T-cells (yet retain IL-10^+^IL-21^+^T-cells), we reconstituted lethally irradiated *Cd4*^*−/−*^ mice with MBM cells from the following donors: *Cd4*^*−/−*^ (70%) + Il10^*−/-*^ *Il21*^*−/−*^ (15%) + 10BiT*-Il21*tRFP (15%), as shown in Fig. 3a. These experimental mice were then challenged with LCMV Cl13, and we proceeded to administer anti-Thy1.1 depletion or IgG2a isotype control antibodies to these groups of mice on days 4 and 6 p.i., before conducting analyses on day 21 p.i. The efficacy in depleting Thy1.1^+^(IL-10^+^)Tfh cells was >90% (Fig. 3b and Supplementary Figure 4A). Of note, we did not observe any significant difference between groups in the total number of effector CD4 T cells responding to LCMV Cl13 infection, nor in the proportion or total number of IL-21^+^ single-producing Tfh cells (Fig. 3b and Supplementary Figure 4A). However, depletion of IL-10^+^IL-21^+^CD4 T cells during the first week of Cl13 infection precluded the development of IL-10^+^IL-21^+^Tfh cells by day 21 p.i. (Fig. 3b and Supplementary Figure 4A), suggesting that early differentiation signals may be necessary for the induction of this unique Tfh subset. Strikingly, depletion of IL-10^+^IL-21^+^ dual-producing CD4 T cells severely diminished the GC reaction, as indicated by the marked fourfold reduction in the proportion and total number of GL-7^+^Fas^+^GC B cells (Fig. 3c). Consistent with abrogated GC B cell responses, T-dependent LCMV-specific antibody titers of the IgG1, IgG2a, and IgG2b isotypes were reduced by ~ 40–60% in MBM mice depleted of IL-10^+^IL-21^+^CD4 T cells (Fig. 3d), which was further associated with impaired control over viral replication (Fig. 3f). Of note, sera levels of T-independent antibody isotypes IgM and IgG3 were similar between experimental groups (Supplementary Figure 4B). Importantly, depletion of IL-10^+^IL-21^+^CD4 T cells also resulted in diminished neutralizing antibody titers (Fig. 3e), and linear regression analyses demonstrated that virus-specific antibody titers inversely correlated with viral load (Supplementary Figure 4C), indicating that the enhanced protection observed in control MBM mice is likely humoral-mediated. Collectively, these data demonstrate that IL-10^+^IL-21^+^ co-producing CD4 T cells are critical for sustaining GC reactions, promoting B cell immunoglobulin class-switching to cytophilic antibody isotypes, and limiting viral replication during chronic LCMV infection.

### Tfh-derived IL-10 supports antiviral humoral immunity

**Fig. 4:**
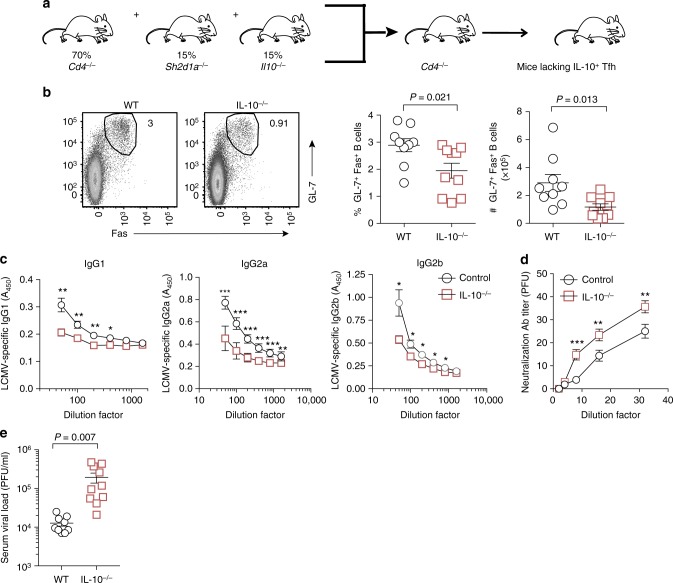
Tfh-derived IL-10 sustains antiviral humoral immunity. **a** Experimental scheme of generating MBM chimeric mice with Tfh-specific IL-10 deletion. **b**–**e** WT and *Il10*^*−/−*^ Tfh MBM chimeric mice were infected with LCMV Cl13. On day 21 p.i., GC reactions and antibody responses were examined. **b** Representative flow plots (left) and scatter graphs (right) showing the frequency and total number of GC B cells from WT and *Il10*^*−/−*^ Tfh MBM chimeric mice. **c** Summary graphs showing serum levels of LCMV-specific IgG1, IgG2a, and IgG2b. **d** Summary graph showing relative antibody neutralization titers for control and *Il10*^*−/−*^ Tfh MBM chimeric mice. **e** Serum viral titers from WT and *Il10*^*−/−*^ MBM chimeric mice as determined by plaque assay. Summary data (mean + /- SEM in **b–e**) are pooled from two independent experiments with five mice/group per experiment. *P < 0.05, **P < 0.01, ***P < 0.0001. Data were analyzed using two-tailed unpaired student’s *t* tests. See also Figure S4

Given that IL-10^+^IL-21^+^Tfh cells exhibited a more robust GC-like Tfh profile as compared with their IL-10^−^IL-21^+^ counterparts (Fig. 2c-f), we hypothesized that IL-10-producing Tfh cells play a critical role in sustaining GC and B cell-secreted antibody responses during chronic viral infection. To specifically assess the importance of GC Tfh-derived IL-10 in regulating humoral immunity during chronic infection, we generated MBM chimeras in which groups of CD4^*−/−*^ mice were lethally irradiated and reconstituted with BM from the following donors: *Cd4*^*−/−*^ (70%) + *Sh2d1a*^*−/−*^ (*Sh2d1a* encodes SAP) (15%) + either *Il10*^*−/−*^ (15%) or WT (15%) (Fig. 4a and Supplementary Figure 4D). In this manner, IL-10 deficiency is selectively restricted to GC Tfh cells, but not other CD4 T-cell subsets. These groups of MBM mice were then infected with LCMV Cl13, and on day 21 p.i. we examined the magnitude of the GC and LCMV-specific antibody responses. Notably, abrogation of IL-10 production by Tfh cells resulted in ~ 2–3-fold decreases in the proportion and number of GL-7^+^Fas^+^GC B cells (Fig. 4b). IL-10 deficiency within the Tfh compartment also had deleterious effects on the Tfh response itself, as MBM chimera mice with *Il10*^*−/−*^ Tfh cells harbored 50% fewer numbers of CXCR5^+^PD-1^hi^Tfh cells (Supplementary Figure 4E**)**. In alignment with these findings, MBM chimera mice with Tfh-specific IL-10 deletion had ~ 40–50% lower levels of LCMV-specific IgG1, IgG2a and IgG2b serum antibody titers (Fig. 4c, d), which strongly correlated with their diminished control over viral replication (Fig. 4e and Supplementary Figure 4G). Collectively, these data demonstrate that Tfh-produced IL-10 is essential for maintaining protective humoral responses during chronic viral infection.

### B cell-intrinsic IL-10R signaling sustains humoral immunity

**Fig. 5:**
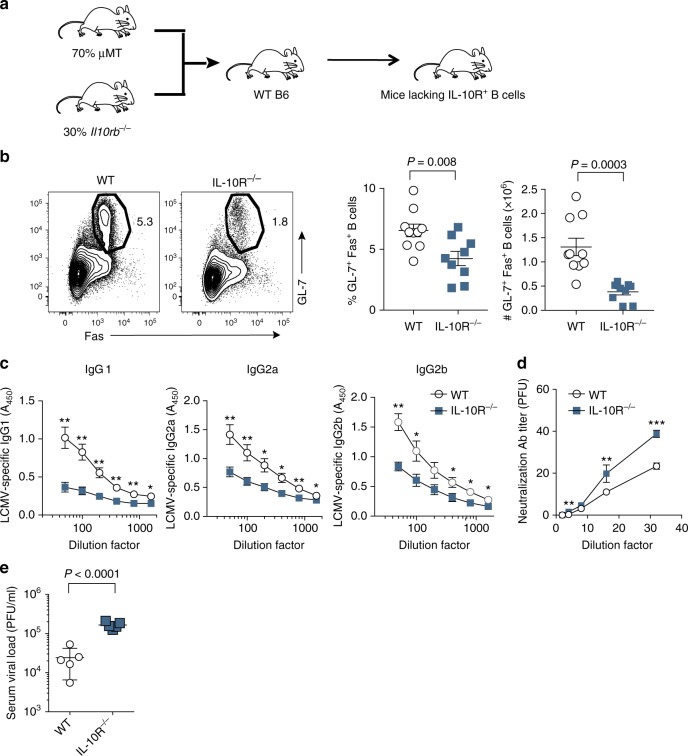
B cell-intrinsic IL-10 signaling is essential for maintaining GC reactions. **a** Experimental scheme of generating MBM chimeric mice with B cell-specific *Il10r* deletion. **b**–**d** WT and *Il10r*^*−/−*^ B cell MBM chimeric mice were infected with LCMV Cl13 and B cell responses were examined on day 21 p.i. **b** Representative flow plots (left) and scatter graphs (right) showing the frequency and total number of GC B cells from WT and *Il10r*^*−/−*^ MBM chimeric mice. **c** Summary graphs showing serum levels of LCMV-specific IgG1, IgG2a, and IgG2b. **d** Summary graph showing relative antibody neutralization titers for control and *Il10r*^*−/−*^ B cell MBM chimeric mice. **e** Serum viral titers from WT and *Il10r*^*−/−*^ mice as determined by plaque assay. Summary data (mean + /- SEM in **b–e**) are pooled from two independent experiments with 4-5 mice/group per experiment. *P < 0.05, **P < 0.01, ***P < 0.0001. Data were analyzed using two-tailed unpaired student’s *t* tests. See also Figure S6

The necessity of Tfh-derived IL-10 in maintaining humoral immunity during LCMV Cl13 infection raised the question as to whether IL-10 is acting in an intrinsic manner on either Tfh cells themselves, or on B cells to reinforce the GC reaction. To test this, a series of MBM chimeras were generated. First, to test the importance of IL-10 signaling in Tfh cells, *Cd4*^*−/−*^ mice were lethally irradiated and reconstituted with BM from the following donors: *Cd4*^*−/−*^ (70%) + *Sh2d1a*^*−/−*^ (15%) + either *Il10rb*^*−/−*^ (15%) *or* WT (15%) (Supplementary Figure 5A). In this manner, Tfh cells in control mice are capable of responding to IL-10 signaling, whereas Tfh cells in chimeric mice reconstituted with *Il10rb*^*−/−*^ BM are not. Second, to test whether B cell-intrinsic IL-10R signaling is required to maintain humoral immunity during chronic infection, we reconstituted lethally irradiated WT mice with BM from *μMT* (70%) + either *Il10rb*^*−/−*^ (30%) *or* WT (30%) mice (Fig. 5a). Groups of MBM chimera mice were infected with LCMV Cl13 and on day 21 p.i., we assessed GC- and LCMV-specific antibody responses. Importantly, selective deficiency of IL-10 signaling in Tfh cells had no appreciable effect on either the magnitude of the GC reaction, the level of LCMV-specific antibody titers, or viral containment (Supplementary Figure 5B–E). These data demonstrate that although Tfh-derived IL-10 is essential for sustaining GC and antibody responses, Tfh cells themselves are indirectly affected by IL-10 signaling. In sharp contrast, selective deficiency of IL-10 signaling in B cells severely impaired the development of GC reactions, as witnessed by two to threefold reductions in the proportion and total numbers of both CXCR5^+^PD-1^hi^Tfh cells and GL-7^+^Fas^+^GC B cells (Fig. 5b and Supplementary Figure 6A). Moreover, the diminished GC reactions in *Il10rb*^*−/−*^ B cell chimeric mice, were accompanied by ~ 50–60% reductions in serum titers of LCMV-specific IgG1, IgG2a, and IgG2b isotypes (Fig. 5c, d), and over a 10-fold increase in sera viral titers (Fig. 5e). Additionally, the relative amounts of circulating IgG1, IgG2a, and IgG2b (but not IgM or IgG3 (Supplementary Figure 6B)) once again inversely correlated with viral load (Supplementary Figure 6C), indicating that the protective effects of IL-10 in controlling viral replication is likely antibody-mediated. Taken together, these data demonstrate that Tfh-derived IL-10 mediates antiviral immunity in a manner that is critically dependent on IL-10 signaling in B cells. Intriguingly, whereas the relative amounts of Bcl-6 and Blimp-1 in GC B cells did not differ between groups (Supplementary Figure 6D), we identified that expression of T-bet, an important orchestrator of B cell responses during chronic LCMV infection^38^, was enhanced by ~20% in GC B cells that retained intact IL-10 signaling (Supplementary Figure 6D).

### IL-27 and type I IFNs regulate IL-10^+^ Tfh development

As our results identified a critical role for IL-10-producing Tfh cells in supporting humoral immunity during chronic viral infection (Fig. 4), we next sought to ascertain the inflammatory signals propagated during persistent LCMV infection that contribute to the formation of this protective cell subset. Our Thy1.1 depletion experiments (Fig. 3) suggested that early differentiation signals may be required for the formation of IL-10^+^Tfh cells. To further assess whether early antigenic or inflammatory signals are responsible for the induction of IL-10^+^IL-21^+^Tfh cells during persistent infection, we transiently depleted CD4 T cells at the time of infection. By day 21 p.i., the proportion of CD44^hi^CD4 T cells was almost restored to that of control mice (Supplementary Figure 7A), although total numbers of effector T-cells were still reduced ~twofold (Supplementary Figure 7A). Intriguingly, early depletion of CD4 T cells largely abrogated the generation of IL-10^+^IL-21^+^Tfh cells despite the formation of IL-21^+^ single-positive Tfh cells being mostly intact (Supplementary Figure 7B). Moreover, as CD4-depletion results in prolonged viremia^39^, our data indicate that antigenic stimulation alone is not sufficient for the development of IL-10^+^IL-21^+^Tfh cells during LCMV Cl13 infection.

**Fig. 6:**
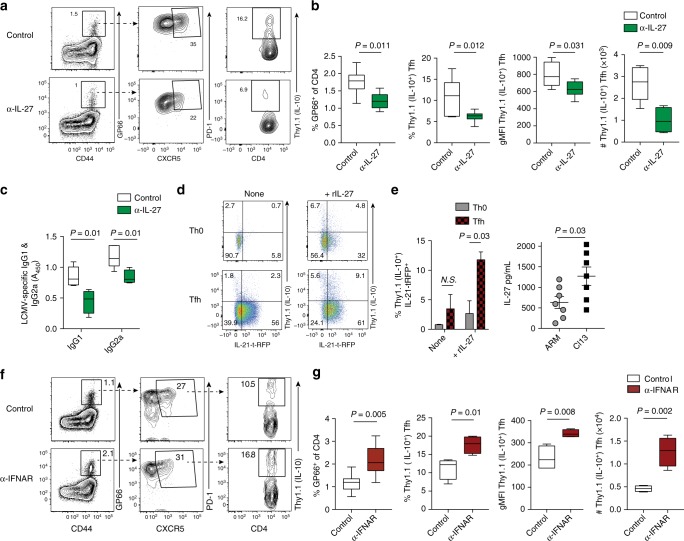
IL-27 and type I IFNs differentially regulate IL-10^+^Tfh development. **a–c** *10BiT-Il21-RFP* mice were infected with LCMV Cl13 and treated with either isotype control or α-IL-27p28 blocking antibodies on days 3 and 9 p.i. **a**, **b** Representative flow plots (**a**) and summary data (**b**) depicting the proportion and total number of splenic Thy1.1 (IL-10)^+^ GP66:I-A^b^-specific Tfh cells from control and α-IL-27p28-treated mice on day 14 p.i. The relative expression (gMFI) of IL-10 (Thy1.1) in Tfh cells from experimental mice is also depicted in (**b**). **c** Summary graph showing serum levels of LCMV-specific IgG1 and IgG2a on day 21 p.i. **d** Naive CD4 T cells were purified and cultured under Th0 or Tfh-polarizing conditions in the presence or absence of recombinant IL-27. Flow plots (left) and summary data (right) showing the proportion of Thy1.1 (IL-10^+^) IL-21-tRFP^+^ cells after 3 days of in vitro culture. **e** WT mice were infected with LCMV Arm or LCMV Cl13. (**e**) Summary graph showing serum levels of IL-27 in experimental mice on day 7 p.i. **f**, **g** *10BiT-Il21-RFP* mice were infected with LCMV Cl13 and treated with either isotype control or α-IFNAR blocking antibodies on days 10, 14, and 18 p.i. **f**, **g** Representative flow plots (**f**) and summary data (**g**) depicting the proportion and total number of splenic Thy1.1 (IL-10)^+^ CXCR5^+^PD-1^+^ GP66:I-A^b^-specific Tfh cells from control and α-IFNAR-treated mice on day 21 p.i. The relative expression of IL-10 (Thy1.1) in Tfh cells from experimental mice is also shown in **g**. Summary data (box and whisker plots showing median (midline) and interquartile range with upper and lower whiskers representing the range of data distribution in **b**, **c**, and **g** or mean + /- SEM in **d**, **e**) are from 3 to 5 mice/group and are representative of two to three independent experiments. *P < 0.05, **P < 0.01, ***P < 0.0001. Data were analyzed using two-tailed unpaired student’s *t* tests. See also Figure S7

One potential explanation for the failure of IL-10^+^IL-21^+^Tfh cells to form after CD4-depletion, is that certain viral-induced inflammatory cytokines necessary for the differentiation of IL-10^+^IL-21^+^Tfh cells may have rapidly declined at the time CD4 T cells began to re-emerge within the host^40^. To test this, we performed in vivo neutralization experiments to block the activity of Tfh-polarizing cytokines IL-6, IL-21, or IL-27, all of which have purported roles in facilitating Tfh differentiation^41–44^. As reported previously, disruption of either IL-6 or IL-21 signaling severely diminished Tfh development (Supplementary Figure 7C). However, we did not observe any consistent differences between groups in the relative proportion of IL-10-producing Tfh cells (Supplementary Figure 7C, D). By contrast, inhibition of the IL-27p28-signaling pathway resulted in ~50% decreases in the total number of GP66:I-A^b^-specific and effector Tfh cells, and of the Tfh cells that formed, we observed a striking 50% decrease in the proportion of IL-10-producing cells (Fig. 6a, b and Supplementary Figure 7E, F). Moreover, Tfh expression of Thy1.1 (IL-10) was decreased on a per cell basis by ~25% when IL-27 was neutralized. These alterations in the Tfh compartment were further associated with reduced serum levels of virus-specific IgG1 and IgG2a (Fig. 6c). Notably, IL-27 has previously been demonstrated to induce IL-10 expression in Th1, Th17, and Tr1 subsets^33,45,46^. To determine whether CD4 intrinsic IL-27 signaling can promote the development of IL-10^+^IL-21^+^Tfh cells, we performed in vitro culture experiments wherein naive CD4 T cells were polarized along a Tfh differentiation pathway in the presence of recombinant IL-27. Notably, addition of recombinant IL-27 to the culture simultaneously enhanced the production of IL-10 and IL-21 in in vitro-skewed Tfh cells (Fig. 6d), indicating a potential CD4-intrinsic role for IL-27 signaling in promoting the formation of this subset. Collectively, our data demonstrate that IL-27 is a potent inducer of IL-10 expression in Tfh cells during chronic infection (Fig. 2g).

Our finding that IL-27 signaling plays an important role in the formation of IL-10-producing Tfh cells during persistent viral infection, coupled to our observation that IL10^+^IL21^+^Tfh cells predominately form during chronic but not acute LCMV infection (Fig. 2), prompted us to investigate whether potential differences in IL-27 availability exists between these two respective infections. To this end, sera levels of IL-27 were assessed at 16 hours, 3 days, and 7 days p.i. in groups of LCMV-Arm and LCMV Cl13-infected mice. Importantly, although sera levels of IL-27 were similar among experimental groups at 16 hours and 3 days p.i. (Supplementary Figure 7G), we identified over a twofold increase in circulating IL-27 levels in Cl13-infected mice on day 7 p.i. (Fig. 6e), which correspondingly is a time point directly preceding the sharp expansion of IL-10^+^IL-21^+^Tfh cells (Fig. 2b). Taken together, our data suggest that overproduction of IL-27 induced during LCMV Cl13 infection, is one of the potential mechanisms responsible for spurring the development of IL-10^+^IL-21^+^Tfh cells.

Finally, persistent LCMV infection is associated with a prolonged type I IFN response^47–49^, which has recently been shown to negatively regulate the induction of protective antibody responses^49–51^. Thus we tested whether blockade of the type I IFN receptor (IFNAR) would enhance the differentiation of IL-10-producing Tfh cells. Strikingly, blockade of IFNAR signaling after the first week of infection resulted in approximately a 60% increase in the proportion of IL-10-producing Tfh cells (Fig. 6f, g and Supplementary Figure 7H, I) which was further associated with a 25% increase in the proportion of GC B cells on day 21 p.i. (Supplementary Figure 7J). Additionally, IL-10 expression was also increased on a per cell basis by ~ 30–40% in GP66:I-A^b^-specific and effector Tfh cells when IFNAR signaling was abrogated (Fig. 6f, g and Supplementary Figure 7H, I). Collectively, these data demonstrate that the expansion and accumulation of IL-10^+^ Tfh cells critically relies on inflammatory and antigenic signals propagated during the early phase of chronic viral infection. Moreover, we identify that IL-27 signaling promotes, while type I IFNs constrain the development of IL-10-producing Tfh cells.

## Discussion

In this study, our single-cell transcriptomics analyses identified a subpopulation of IL-10-expressing CD4 T cells that displayed a robust Tfh signature. This finding is rather unexpected, as Th1 and Tr1 cells are implicated as being the predominant sources of IL-10 during chronic viral and parasitic infections^4,52–54^. Importantly, our study demonstrates that a stable population of IL-10^+^Tfh cells develop during chronic but not acute LCMV infection, and that their formation is essential to sustain humoral immunity in the face of persistent antigenic exposure.

While, the transcriptional program and environmental cues important for Tfh differentiation are becoming increasingly well-defined, the precise molecular mechanisms by which Tfh cells sustain humoral immunity during chronic infections is a matter of ongoing investigation. To date, the B cell “helper” effect of IL-21 has garnered most of the attention. However, the role of other Tfh-derived cytokines in regulating humoral responses remains less well-explored. In this study, we have identified a unique subpopulation of Tfh cells that co-express IL-10 and IL-21 during persistent infection. Moreover, selective depletion of IL10^+^IL-21^+^CD4 T cells revealed that the coordinate production of IL-10 and IL-21 by CD4 T cells responding to chronic LCMV infection is essential for sustaining humoral immunity. As T-cell-derived IL-10 is generally considered to be an immunosuppressive cytokine that limits viral control^4,6–8^, our finding that Tfh-derived IL-10 positively instructs GC reactions and antiviral immunity is a rather unexpected discovery.

Notably, our study identifies that distinct environmental and inflammatory signals propagated during chronic but not acute LCMV infection promote the formation of this unique IL-10^+^IL-21^+^Tfh subset. Intriguingly, our data also demonstrate that a small population of IL-10^+^IL-21^+^Tfh cells also develop during PR8-influenza. Thus, the development of IL-10^+^IL-21^+^Tfh cells appears to be context-dependent, and it is possible that these cells may play a role in regulating humoral responses during various immunological settings. Importantly, however, our data additionally indicate that the formation of IL-10^+^IL-21^+^Tfh cells during influenza appears to be rather transient. Moreover, our unpublished observations indicate that the majority of IL-10-producing CD4 T cells with a CXCR5^+^PD-1^+^ phenotype co-express Foxp3 during PR8 infection, suggesting that this minor IL-10^+^ subset may be predominantly comprised of TFR cells. This observation is consistent with two recently published reports identifying that TFR cells significantly expand after viral clearance, including both PR8 influenza and LCMV-Arm infections^55,56^. While the expansion of TFR cells during PR8 was found to be essential to prevent autoreactive B cell responses^55^, this latter study unexpectedly demonstrated that TFR-derived IL-10 can actually promote GC B cell differentiation^56^. However, whether TFR-derived IL-10 has any impact on B cell-secreted antibody responses or viral control remains unclear.

By contrast, our study demonstrates that only a small proportion (<15%) of IL-10^+^ CXCR5^+^PD-1^+^CD44^hi^CD4 T cells co-express Foxp3 during chronic LCMV infection, and that Foxp3 expression is undetectable in the GP66-specific Tfh compartment, indicating the absence of the TFR lineage among virus-specific CD4 T cells. This observation is in agreement with a previous report that identified that GC Tfh and TFR subsets are generated from distinct TCR repertories, with Tfh cells expressing antigen-responsive TCRs to promote antibody responses whereas TFRs appear to express potentially autoreactive TCRs in order to suppress autoimmunity^57^. Our data additionally demonstrate that IL-21 is not highly expressed in Foxp3^+^TFR cells during LCMV Cl13 infection, further indicating that IL-10^+^IL-21^+^Tfh cells are distinct from the TFR lineage.

Given our findings that Tfh-derived IL-10 is essential for promoting antiviral humoral immunity, it was of interest to determine the molecular signals required for the development of IL-10^+^Tfh cells. Our IL-10^+^IL-21^+^ CD4-depletion experiments indicate that early inflammatory signals propagated during the first few weeks of LCMV Cl13 infection are necessary for the formation of IL-10^+^IL-21^+^Tfh cells. In alignment with this idea, the Tfh-skewing cytokine IL-6 is reportedly produced in a biphasic fashion^40^. Thus, particular cytokines important for Tfh differentiation may have been absent at the time CD4 T cells began to re-emerge within the host following transient depletion of either CD4 or IL-10^+^IL-21^+^CD4 T cells. Therefore, we explored a potential role for the cytokines IL-6, IL-21, and IL-27 in the early induction of IL-10-producing Tfh cells during LCMV Cl13 infection. Our in vivo neutralization experiments suggest that IL-6 and IL-21 largely impact the magnitude of the Tfh response, whereas IL-27 appears to be one of the key cytokines involved in the induction of IL-10^+^Tfh cells during chronic infection. However, it remains to be determined whether other cytokines, such as IL-12 or IFN-γ, both of which have purported roles in promoting Tfh differentiation^58–60^, further contribute to the development of IL-10^+^Tfh cells. Intriguingly, our in vitro Tfh polarization experiments indicate that direct IL-27 signaling may promote the development of IL-10^+^Tfh cells, although it remains unclear as to whether this induction process can occur in vivo. Additionally, although the pathways downstream of IL27R signaling that promote the differentiation of IL-10^+^Tfh cells remain relatively unexplored, our RNA-seq analyses potentially indicate that c-Maf, a known downstream target of IL-27 signaling^33^, may be associated with this induction. Notably, several studies have identified that c-Maf plays a critical role in facilitating Tfh differentiation^61,62^ as well as in promoting IL-10 expression in various CD4 T-cell populations^32,63^. Collectively, our data indicate that IL-27 appears to be one of the important cytokines in promoting the formation of IL-10^+^Tfh cells during LCMV Cl13 infection, although it is possible that other cytokines may also contribute to the development of this protective subset.

Intriguingly, our study has also identified that type I IFNs suppress the formation of IL-10^+^Tfh cells during chronic infection. Several recent studies have demonstrated an inhibitory role for type I IFNs in the development of neutralizing antibody responses during persistent viral infection^49–51^. In these scenarios, the inhibitory effects of type I IFNs occurred in a B cell-extrinsic fashion and involved IFNAR signaling in multiple cell types, including myeloid cells, dendritic cells, and T cells. Interestingly, previous studies have also identified that IFNAR blockade during chronic infection results in enhanced viral clearance^47,48^. Our study further expands on these studies and demonstrates that type I IFNs may also impede the development of IL-10^+^Tfh cells. Hence, our results may also indicate a potential mechanism by which type I IFNs function to impede the development of protective antibody responses during persistent infection.

Collectively, our work highlights a previously unrecognized circuit wherein Tfh-secreted IL-10 maintains GC reactions and supports the production of virus-specific antibodies. Importantly, our study also identifies that B cell-intrinsic IL-10 signaling is pivotal for sustaining humoral responses during chronic viral infection. This finding is consistent with a recent report that demonstrated IL-10R-signaling in B cells is essential for the induction of GC reactions and humoral immunity during *Plasmodium* infection^64^. Thus, the necessity of B cell-intrinsic IL-10R signaling in sustaining humoral immunity may be a generalizable feature of chronic infections. In summary, our study ascertains that Tfh-derived IL-10 is a key positive regulator of GC reactions and antibody responses during chronic infection. Thus, therapeutic strategies aimed at enhancing Tfh production of IL-10 may serve to bolster humoral immunity during persistent viral infection.

## Methods

### Mice and viruses

All mice were bred and maintained in a closed breeding facility, and mouse handling conformed to the requirements of the Institutional Animal Care and Use Guidelines of Medical College of Wisconsin. C57BL/6, *Il10r*^*−/−*^*, Il10*^*−/−*^*, Il21*^*−/−*^, and *Cd4*^*−/−*^ mice were purchased from Jackson Laboratory (Bar Harbor, ME). IL-10 and IL-21 double-reporter mice were generated by cross-breeding IL-21tRFP mice^12,25^ with ten BiT mice (kindly provided by Dr. Casey Weaver, University of Alabama at Birmingham, AL). All mice used in accordance with animal protocols approved by the Medical College of Wisconsin Institutional Animal Care and Use Committee. LCMV Armstrong (Arm) was intraperitoneally injected into mice (2 × 10^5^ PFU/ mouse) to establish an acute infection. LCMV clone 13 (Cl13) was intravenously injected into mice (2 × 10^6^ PFU/mouse) to establish chronic infection. Both strains of viruses were prepared by a single passage on BHK­21 cells, and viral titers were determined by plaque formation assay on Vero cells.

### Flow cytometry

Mouse splenocytes were harvested, subjected to red blood cell lysis, washed, and stained as previously described^12^. Staining with GP66:A-^b^ PE tetramer (NIH tetramer core facility) was performed at room temperature for 1 h. Transcription factor staining was performed using a Foxp3 staining buffer set (eBioscience, CA). To detect Tfh cells, CXCR5 was stained for using rat anti-mouse CXCR5 antibody (BD Bioscience, CA), followed by incubation of a secondary biotin–SP-conjugated Affinipure F(Ab’)2 goat anti-rat IgG (Jackson Immunoresearch, PA). At the last step, cells were stained with streptavidin–APC (Invitrogen, CA) together with other surface markers. In some experiments, anti-CXCR5 APC or APCcy7 antibody conjugates were used. All antibodies used in this study are listed in Supplemental Table 1. All flow cytometry data were acquired on an LSRII (BD Biosciences, CA) and analyzed by FlowJo (Treestar, OR).

### In vitro CD4 polarization assays

Twenty-four-well plates were coated with anti-CD3 antibodies (2 ug/mL; Biolegend) for 2 h at 37 degrees. Naive CD4 T cells from 10Bit *IL-21*-tRFP reporter mice were purified using the EasySep CD4 T cell isolation kit from Stemcell technologies, and 1 × 10^6^ cells were plated per well. Cells were stimulated for 3 days at 37 degrees in the presence of anti-CD28 antibodies (2 µg/ml; Biolegend). Th0 cells were cultured in the presence of 20 ng/mL IL-2. Tfh polarization was achieved by adding 50 ng/mL recombinant IL-6 and anti-IFN-γ and anti-IL-4-neutralizing antibodies (1µg/mL each; Biolegend). Some of the Th0 and Tfh-polarized cells also received recombinant IL-27 (25 ng/mL) at the start of the culture. After 3 days of culture, cells were stimulated with PMA and Ionomycin for 1 h, at which point monensin was added to the culture wells, and cells were then stimulated for an additional 4 h. Polarized CD4 T cells were then assessed for Thy1.1 (IL-10) and IL-21-tRFP expression by flow cytometry.

### Mouse cytokine assay

Serum samples were obtained from LCMV Armstrong and LCMV Cl13-infected WT mice at 16 h, 3 days, and 7 days post infection. Serum samples were sent to Eve Technologies (Calgary, AB), and analyzed using a multiplex cytokine array.

### ELISA for LCMV-specific antibody

To quantify LCMV-specific antibodies, the Cl13-infected BHK cell lysates were used to coat the plate overnight and blocked with 3% BSA/PBS/0.05% Tween 20^1^. After 1 h of blocking, sera from infected mice were serially diluted and incubated on the plate for 90 min. Subsequently antibody detection was performed for IgM, IgG1, IgG2a, IgG2b, and IgG3 isotypes using HRP conjugated anti-mouse antibodies (Southern Biotechnologies, AL) and relative absorption was measured.

### LCMV neutralization assay

Detection of neutralizing activity against LCMV in mouse sera was determined using a focus-forming assay, according to a previously published protocol^65^. In brief, diluted sera were UV irradiated and incubated with 60 PFU of LCMV-CL13 for 90 min at 37 °C. Then MC57G mouse fibroblast cells were added to each well and incubated for 2 to 3 h. Thereafter, 1% methylcellulose was added, and the cells were placed in the incubator. After 48 h of incubation, cells were fixed with 4% formaldehyde in PBS followed by permeabilization with 1% Triton X-100. Foci were visualized by staining with the anti-LCMV nucleoprotein antibody (VL-4, Bio X Cell, NH).

### Administration of biologics

To deplete CD4 T cells, mice received i.p. injections of 500 µg anti-CD4 antibody (clone GK1.5 from BioXCell, NH) or IgG2a isotype control 1 day before LCMV Cl13 infection and on the two consecutive days following infection. Anti-mouse Thy1.1 (clone 19E12 from BioXCell, NH) antibodies were administered on days 4 and 6 post infection to deplete Thy1.1-expressing cells. In all cases, the depletion efficacy was confirmed via flow cytometry. For IL-27 neutralization experiments, mice were treated on days 3 and 9 p.i. with either 500 µg IgG2a isotype control or 500 µg anti-IL-27p28 blocking antibodies (MM27.7B1; as previously described^66^. For in vivo blockade of type I IFN signaling, mice were treated with either 500 µg of MOPC isotype control (BioXcell) or 500 µg of anti-IFNAR (MAR1-5A3; (BioXcell)) blocking antibodies on days 10, 14, and 18 p.i.

### Mixed bone marrow (MBM) chimeras

For MBM chimera experiments, recipient mice were irradiated with 6.5 and 5.5 Gy separated by 8 h. Bone marrow from various donor mice (as depicted in Figures) were mixed at the indicated ratios, and a total of ~ 6 × 10^6^ cells were transferred i.v. Mice were maintained on oral sulfamethoxazole for 2 weeks. Chimerism was assessed at 7 weeks in peripheral blood using congenic markers. Chimerism in the CD4 T cell compartment in mice reconstituted with bone marrow from *Cd4*^*−/−*^ mice, *Sh2d1a*^*−/−*^ mice, and either *Il10*^*−/−*^ or WT mice was ~55% *Sh2d1a*^*−/−*^ and 45% either *Il10*^*−/−*^ or WT amongst 20 experimental mice in two independent experiments. Chimerism in the B cell compartment in mice reconstituted with bone marrow from *μMT* mice + bone marrow from either *Il10rb*^*−/−*^ *or* WT mice was greater than 93%. Experimental MBM mice were infected with LCMV Cl13 at 8 weeks post reconstitution.

### Single-cell RNA sequencing

Thy1.1^+^ (IL-10^+^) GP_61-80_-specific CD4 T cells were FACS-sorted from LCMV Cl13-infected mice on day 16 p.i. and were loaded on the Chromium Controller (10x Genomics). Single-cell RNA-seq libraries were prepared using the Chromium Single Cell 3′ v2 Reagent Kit (10x Genomics) according to manufacturer’s protocol. Libraries were loaded onto an Illumina NextSeq with the NextSeq 500/550 High Output Kit v2 (150 cycles) (FC-404-2002, Illumina) with the following conditions: 26 cycles for read 1, 98 cycles for read 2, and 8 cycles for i7 index. Python Run Downloader (Illumina) was used to download raw sequencing data. Cell Ranger (10x Genomics) functions mkfastq and count were used to demultiplex the sequencing data and generate gene-barcode matrices, respectively. All scRNA-seq analysis was performed in R (version 3.4.0) using the package Seurat (version 2.2.0)^67^. Number of genes detected per cell, number of UMIs, and percent mitochondrial genes were plotted, and outliers were removed (number of genes over 2500, number of UMIs over 8000, and percent mitochondrial genes over 0.08) to filter out doublets and dead cells, leaving 677 of the original 691 cells. Cell cycle genes were regressed out using a list of S phase genes and G2/M phase genes^67,68^. Principal component analysis was performed, and the top three most statistically significant principal components were used for *t*-SNE analysis, with 2000 iterations and a perplexity parameter of 30.

### Bulk RNA sequencing

Naive (CD44^lo^) CD4 T cells, IL-21^+^ single-positive Tfh cells (CD44^hi^ CXCR5^+^ PD-1^hi^), and IL-10^+^IL-21^+^ double-positive Tfh cells were FACS-sorted from the spleens of LCMV Cl13-infected *10BiT-Il21*tRFP double-reporter mice at day 18 p.i. RNA-seq libraries were prepared using SMART-seq technology^69^ and sequenced on an Illumina NextSeq. Reads were aligned to mm9 transcriptome using TopHat (version 2.1.1) and differential gene expression was determined using Cuffdiff (Cufflinks version 2.2.2)^70^.

### Microscopy

Spleens were fixed with periodate-lysine paraformaldahyde and then snap-frozen in OCT tissue-freezing solution and stored at −80 °C. Tissues were cut into 7-µm sections, placed onto superfrost glass slides and stored at −80 °C until staining. Prior to immunostaining, tissues were rehydrated and blocked with 2% BSA, 5% goat serum, and 2% fetal calf serum. Reagents used to stain sections are listed in Supplementary Table 1. Images were obtained using a Nikon TI2-E inverted microscope at 40x magnification and Imaris software (Bitplane) version 9 was used to prepare images.

### Statistical analyses

Statistical tests were performed using Graphpad Prism 7. *P*-values were calculated using two-tailed unpaired student’s *t* tests, unless otherwise indicated.

## Electronic supplementary material

Supplementary Information

Reporting Summary

## Data Availability

The bulk RNA-seq and single-cell RNA-seq data have been deposited in the GEO database with the accession code GSE111027. A reporting summary for this Article is available as a Supplementary Information file. All other relevant raw data are available from the corresponding author upon request.
