# Supplementary material for: Single-cell RNA sequencing unveils an IL-10-producing helper subset that sustains humoral immunity during persistent infection
Source: Nat Commun. 2018 Nov 28;9:5037. doi: 10.1038/s41467-018-07492-4 (PMC6261948; doi:10.1038/s41467-018-07492-4)
Supplement: Supplementary file 1 — Supplementary Information [file 41467_2018_7492_MOESM1_ESM.pdf]

Single-cell RNA sequencing unveils an IL-10-producing T follicular helper subset that sustains humoral immunity during persistent infection

Xin et al.

Supplementary Material

Xin et al. Supplementary Figure 1

A.

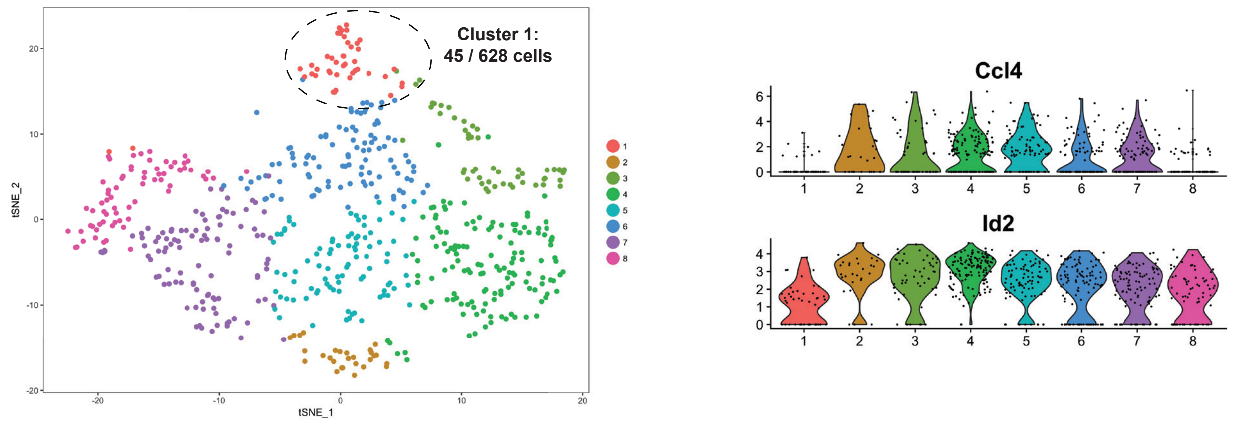

B.

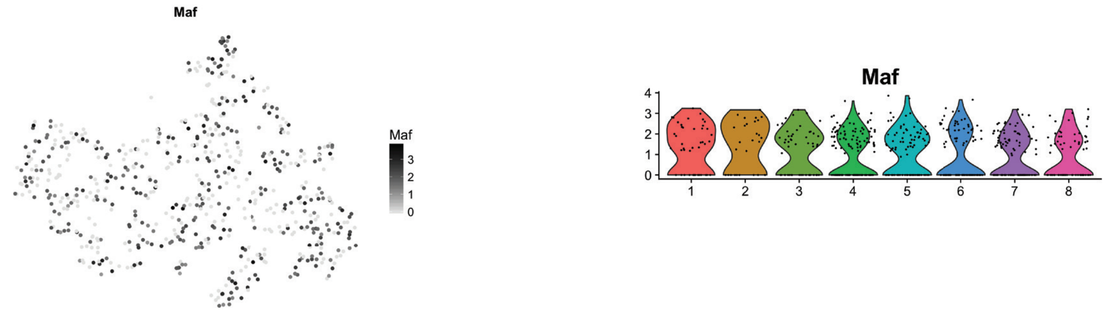

C.

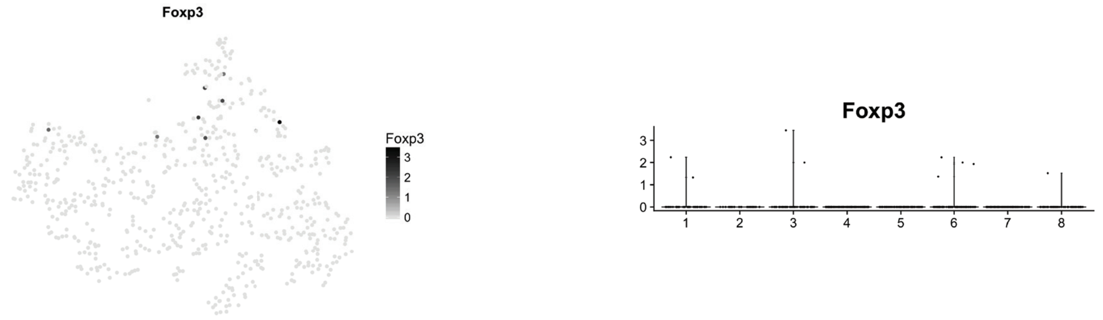

D.

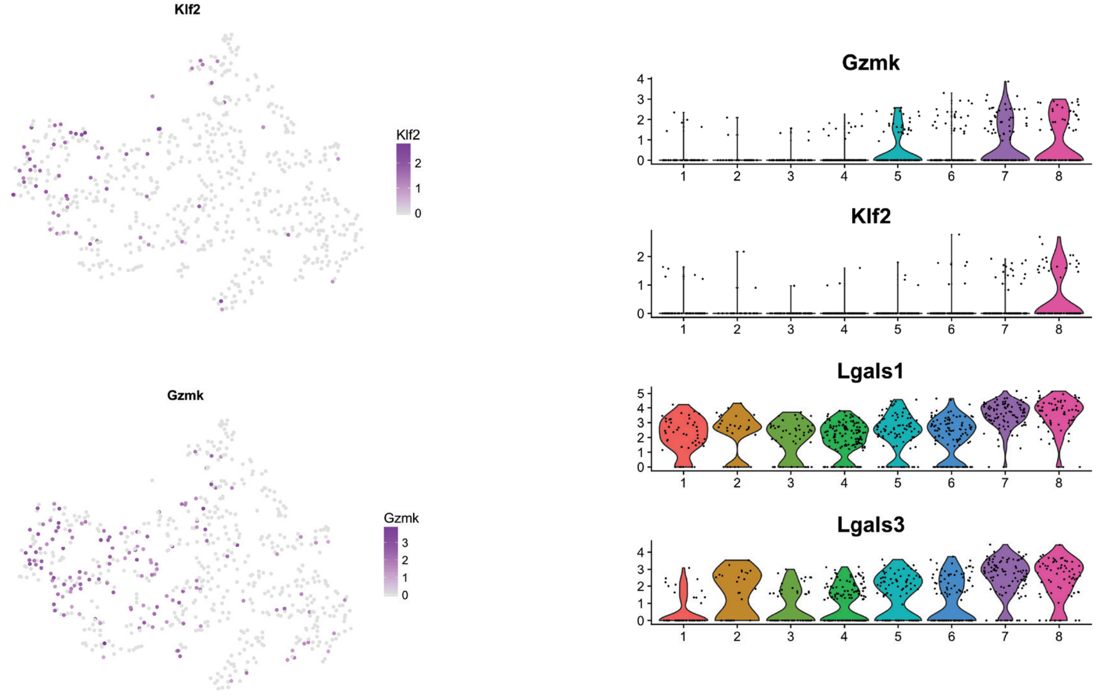

Supplementary Figure 1. scRNA-seq identifies heterogeneity among IL-10$^+$ CD4 T cells responding to LCMV Cl13 infection. Related to Figure 1.

(**A**) *t*-SNE projection of 628 IL-10-producing CD4 T cells from LCMV Cl13-infected mice at day 16 p.i. colored by cluster. Violin plots depicting expression of Th1-associated genes (*Ccl4*, *Id2*), (**B-C**) *t*-SNE plots (left) and violin plots (right) showing relative expression of Maf and Foxp3 in each cell. (**D**) *t*-SNE plots (left) and violin plots (right) depicting expression of genes (*Klf2, Gzmk, Lgals1, and Lgals3*).

Xin et al. Supplementary Figure 2

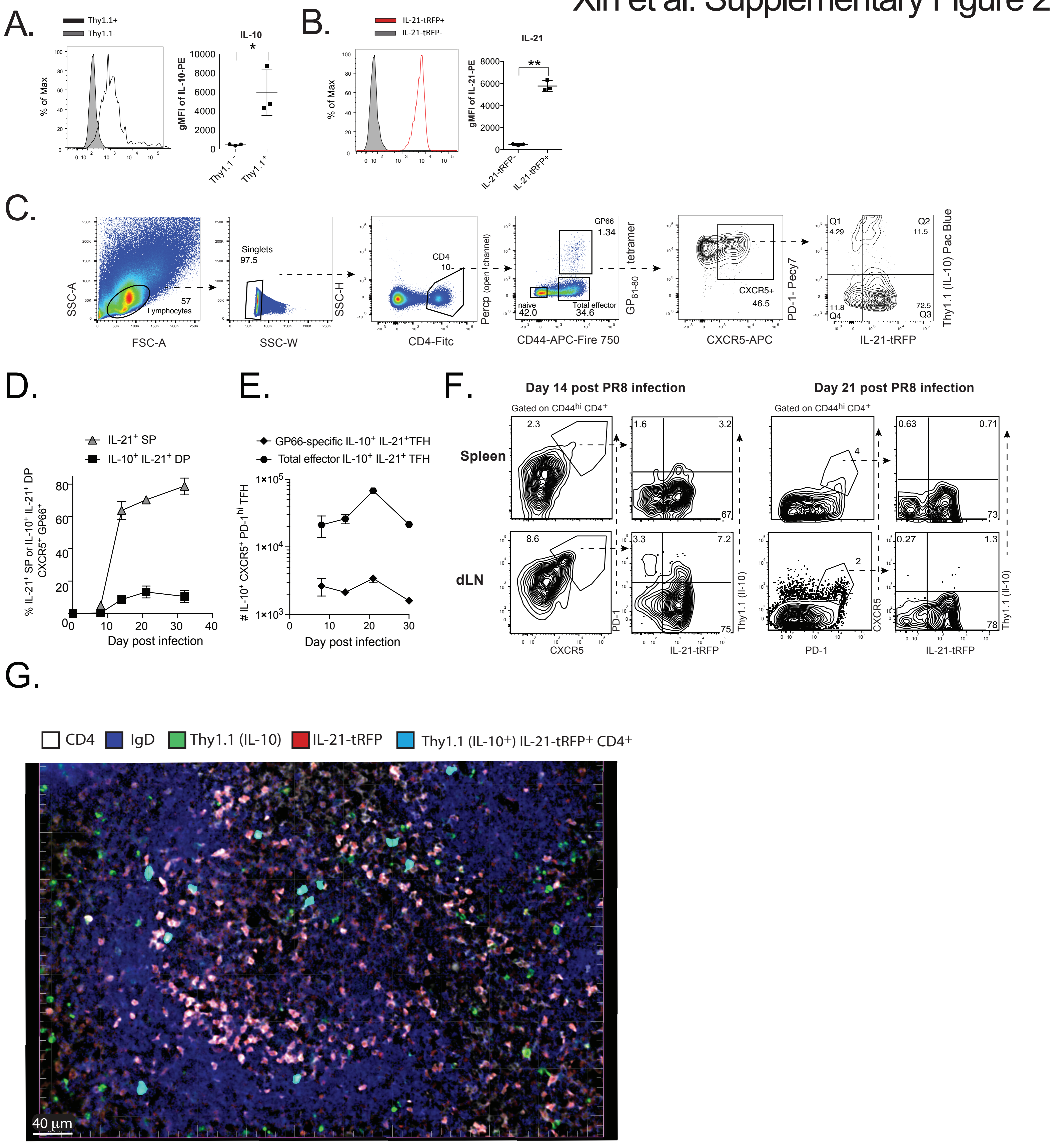

Supplementary Figure 2. IL-10^{+}Tfh cells are a distinct population of T helper cells. Related to Figure 2. (A-B). Thy1.1^{+}IL-21-tRFP^{+} and Thy1.1^{-} IL-21-tRFP^{-} CD44^{hi} CD4 T cells were sort-purified from LCMV Cl13-infected *10BiT-Il21-RFP* reporter mice on day 14 p.i. and stimulated with PMA and Ionomycin for 5 hours in the presence of Brefeldin A. Cells were then stained for intracellular IL-10-PE (JES5-16E3) or IL-21 using mouse IL-21R/human Fc-chimera (IL-21R-Fc) and anti-human Fc-PE. Representative histograms and summary data showing the relative expression of intracellular IL-10 (**A**) or IL-21 (**B**). (**C-E, G-K**). *10BiT-Il21-RFP* mice were infected with LCMV Cl13. (**C-D**) Representative flow plots from day 21 p.i. (C) and summary kinetics (D) showing the proportion of IL-21^{+} and IL-10^{+}IL-21^{+}GP66:I-A^{b} –specific Tfh cells during LCMV Cl13 infection. These flow plots represent the gating strategy used to assess Tfh responses in LCMV-infected mice in Figures 2A-D, 6A-B,F-G, Supplemental Figures 3A-C, 7B-F and H-I). (**E**) Summary graph showing the total numbers of IL-10^{+}IL-21^{+}effector and GP66:I-A^{b} –specific Tfh cells during LCMV Cl13 infection. (**F**). *10BiT-Il21-RFP* mice were infected intranasally with PR8 influenza. The proportion of Thy1.1(IL-10^{+})IL-21-tRFP^{+}Tfh cells were analyzed in the spleen and draining lymph nodes (dLN) on days 14 and 21 p.i. (**G**). Microscopy of GCs from *10BiT-Il21-RFP* mice on day 21 p.i.; stained for IgD(blue) CD4(white) Thy1.1(green), and IL-21(red). Teal pseudocolor represents CD4 T cells that have high staining intensity for both Thy1.1 and IL-21. Data in (A,F) are representative of at least 2 independent experiments and were analyzed using two-tailed unpaired student's t tests.* P<0.05, ** P<0.01, *** P<0.0001.

Xin et al. Supplementary Figure 3

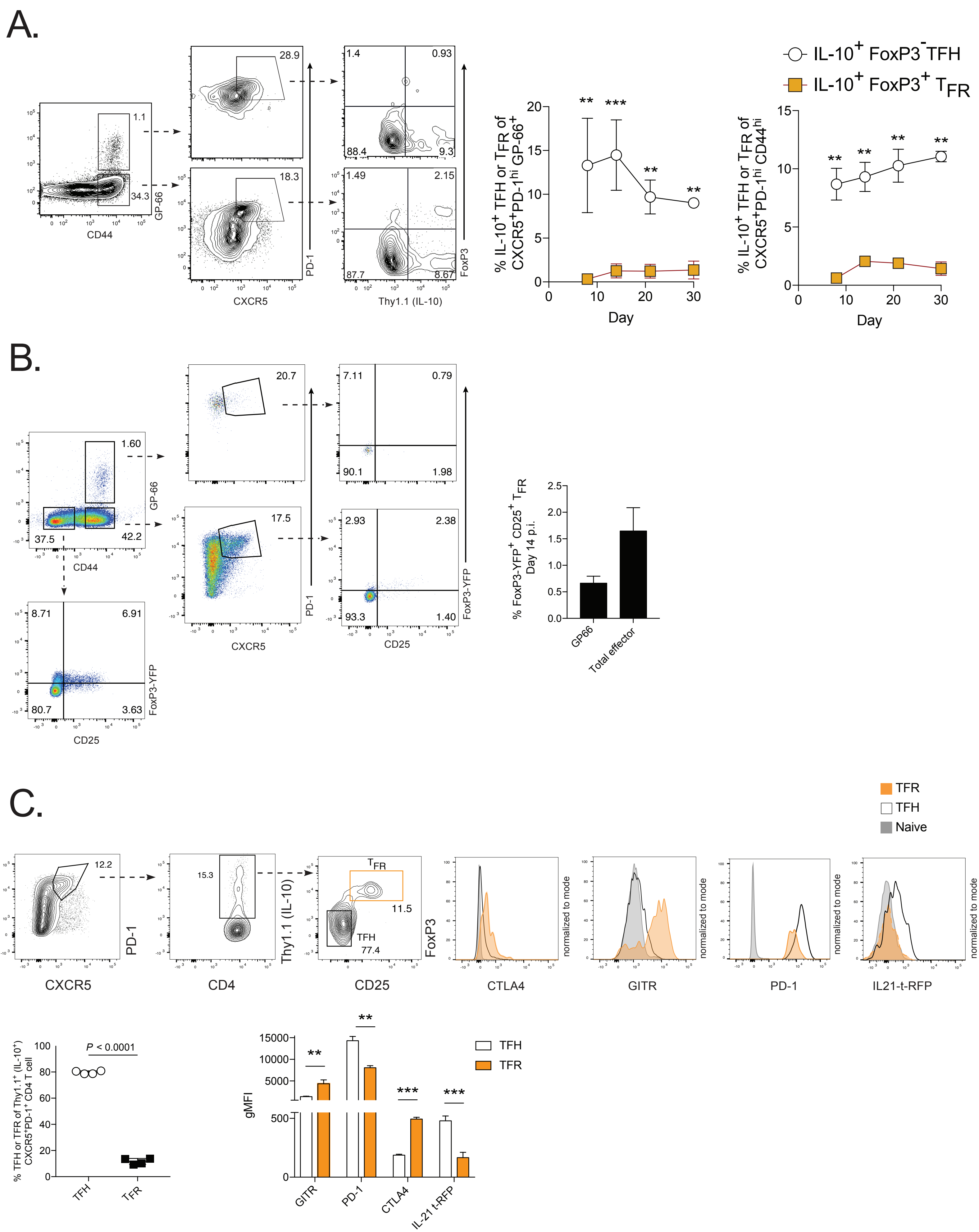

Supplementary Figure 3. Foxp3 is undetectable in virus-specific IL-10$^+$ Tfh cells, but is expressed in a minor population of IL-10$^+$CXCR5$^+$PD-1$^+$ total effector CD4 T cells. (**A and C**) *10BiT-Il21-RFP* mice were infected with LCMV Cl13. (**A**) Representative flow plots depicting Foxp3 expression in Thy1.1$^+$ (IL-10$^+$) and Thy1.1$^-$ (IL-10$^-$) CXCR5$^+$PD-1^{hi} CD44^{hi} GP66:I-A^b –specific and total effector Tfh cells on day 21 p.i. (**B**) Foxp3-YFP reporter mice were infected with LCMV Cl13 and splenic Tfh cells were assessed for Foxp3 expression on day 14 p.i. Representative flow plots (left) and summary data (right) showing the proportion of GP66:I-A^b –specific and total effector Tfh cells co-expressing Foxp3-YFP and CD25. (**C**) Representative flow plots showing the relative expression of CTLA4, GITR, PD-1, and IL-21-tRFP in Thy1.1$^+$ (IL-10$^+$) splenic TFH and TFR cells on day 21 p.i. Data (Mean+/- S.D.) in A and C are from 3-5 mice per time point and are representative of 2-3 independent experiments. Data (Mean +/- S.D.) in B are from 3 mice and are representative of 2 independent experiments. Data in (A,C) are representative of at least 2 independent experiments per time point and were analyzed using two-tailed unpaired student's t tests.* $P<0.05$, ** $P<0.01$, *** $P<0.0001$.

Xin et al. Supplementary Figure 4

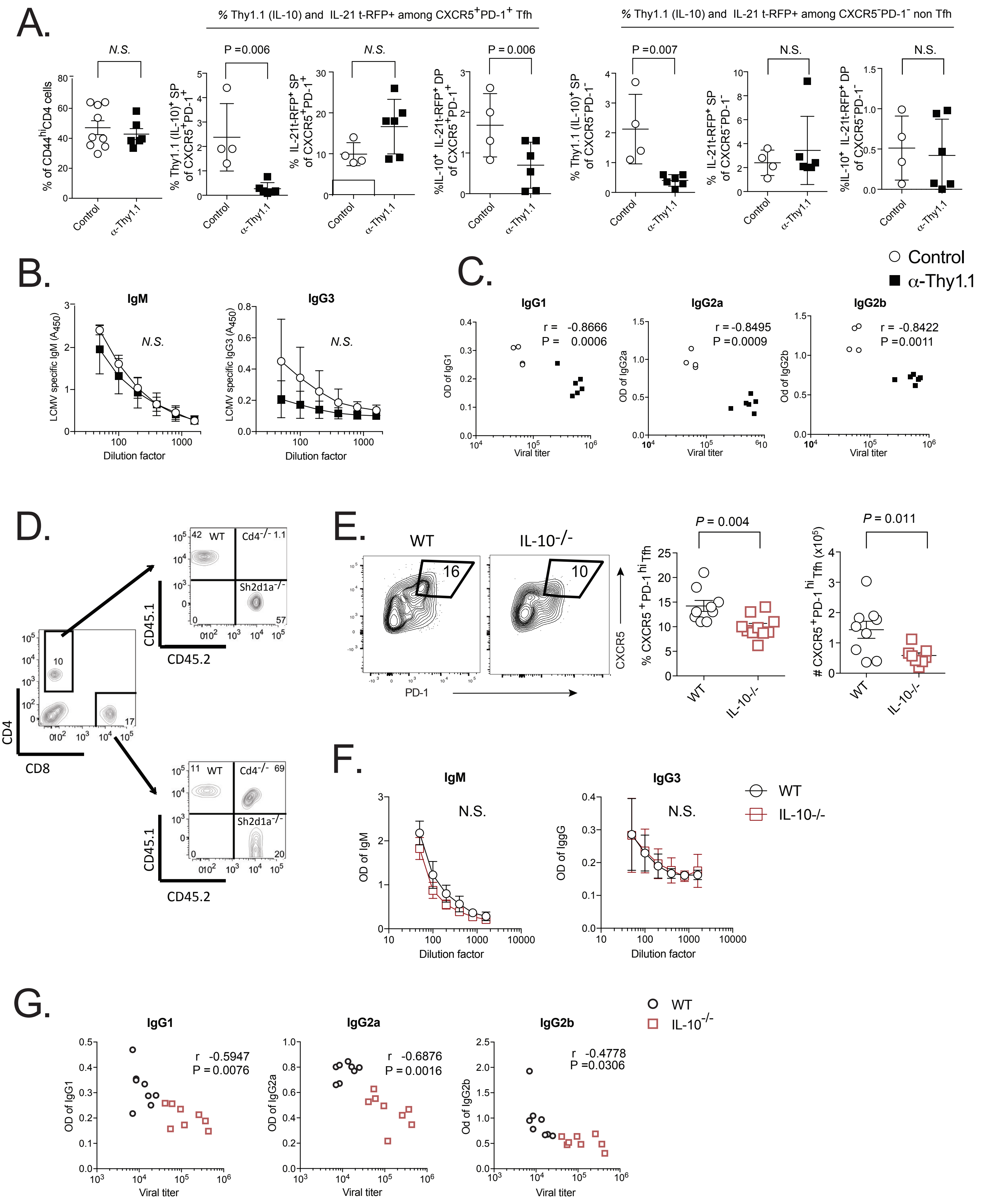

Supplementary Figure 4. IL-10$^+$IL-21$^+$CD4 T-cells and Tfh-derived IL-10 are critical to sustain humoral immunity during LCMV Cl13. Related to Figures 3 and 4.

(**A-C)** MBM chimera mice were generated as described in Figure 3. MBM mice were then infected with LCMV Cl13 and treated with either Thy1.1 depletion or isotype control antibodies on days 4 and 6 p.i. (**A**) Summary graphs showing the proportion of CD44^{hi} effector CD4 T cells (far left) or the proportions of Thy1.1$^+$(IL-10$^+$) SP, IL-21-tRFP$^+$ SP, and Thy1.1$^+$ IL-21-tRFP$^+$ DP Tfh (middle) and non-Tfh cells (right) in experimental mice on day 21 p.i. (**B**) Summary graphs showing the relative titer of virus-specific IgM and IgG3 in the sera of experimental mice (**C**) Summary data showing correlation between relative antibody titers and viremia in experimental mice (**D**) Representative flow plots displaying MBM chimera reconstitution data at 7 weeks post transplant. Reconstitution data is shown for the CD4 T cell compartment (top) and CD8 T cell compartment (bottom). Recipients received bone marrow from the following donors: CD45.1/CD45.2 *Cd4*$^{-/-}$ mice (70%) CD45.2/CD45.2 *Sh2d1a*$^{-/-}$ mice (15%) and CD45.1/CD45.1WT mice (15%). (**E-G**) WT and *Il10*$^{-/-}$ Tfh MBM chimeric mice were infected with LCMV Cl13. On day 21 p.i., GC reactions were examined. (**E**) Representative contour plots (left) and scatter graphs (right) showing the frequency and total number of CXCR5$^+$ PD-1^{hi} Tfh cells from WT and *Il10*$^{-/-}$ Tfh MBM chimeric mice. (**F**) Summary graphs showing the relative titer of virus-specific IgM and IgG3 in the sera of experimental mice (**G**) Summary data showing correlation between relative antibody titers and viral burden in experimental mice. Data were analyzed using two-tailed unpaired student's t tests (Supplemental Figure 4A-B,E-F) or linear regression (Supplemental Figure 4 C,G). *P<0.05, ** P<0.01, *** P<0.0001, (N.S.= not significant).

Xin et al. Supplementary Figure 5

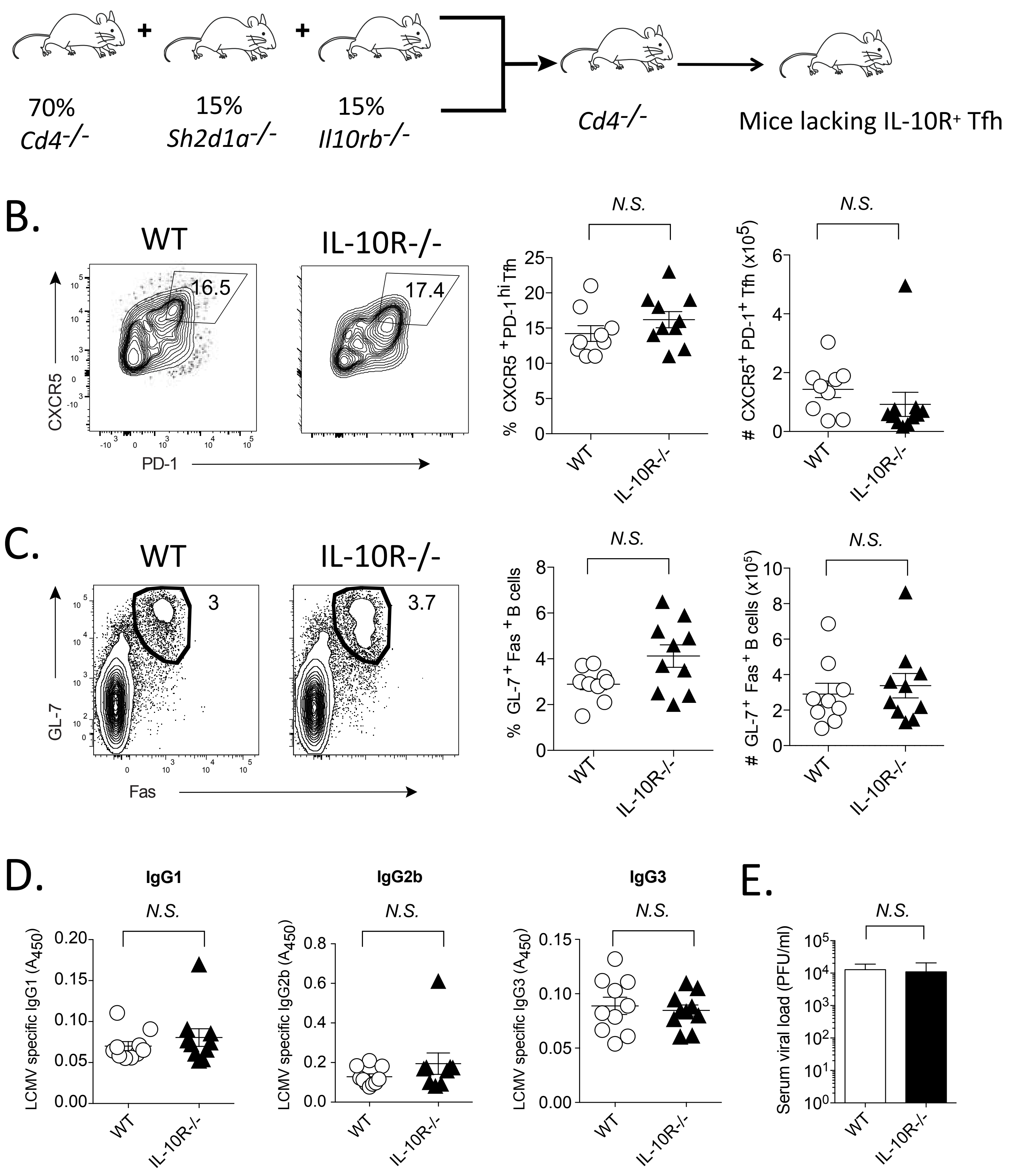

Supplementary Figure 5. Tfh intrinsic IL-10R signaling is dispensable for humoral immunity during persistent infection. Related to Figure 5.

(**A**) Experimental scheme of generating MBM chimeric mice with Tfh cell-specific IL-10R deletion. (**B-E**) WT and *Il10R*$^{-/-}$ Tfh MBM chimeric mice were infected with LCMV Cl13. On day 21 p.i., GC reactions and antibody responses were examined. (**B-C**) Representative contour plots (left) and scatter graphs (right) showing the frequency and total number of Tfh cells (B) and GC B cells (C) from WT and *Il10R*$^{-/-}$ Tfh MBM chimeric mice. (**D**) Summary graphs showing serum levels of LCMV-specific IgG1, IgG2b, and IgG3. (**E**) Serum viral titers from WT and *Il10*$^{-/-}$ MBM chimeric mice as determined by plaque assay and shown in bar graphs. Summary data (mean +/- SEM in B-E) are pooled from 2 independent experiments with 5 mice per group per experiment and were analyzed using unpaired student's t tests. (N.S.= not statistically significant).

Xin et al. Supplementary Figure 6

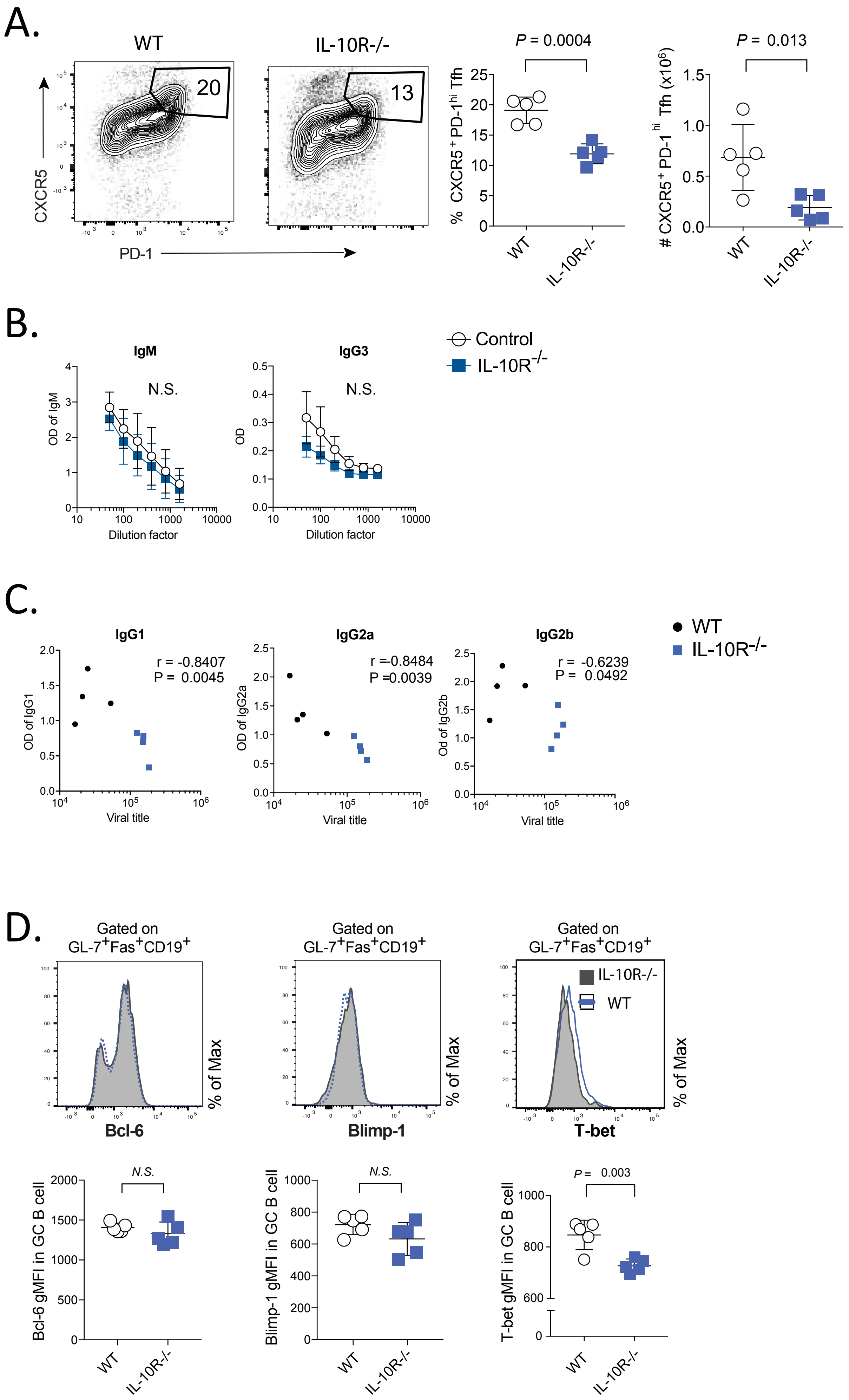

Supplementary Figure 6. B cell intrinsic IL10R-signaling is critical to sustain GC reactions during chronic viral infection. Related to Figure 5.
(**A-B**) WT and *Il10R*$^{-/-}$ B cell MBM chimeric mice were infected with LCMV Cl13 and GC responses were examined on day 21 p.i. (**A**) Representative contour plots (left) and scatter graphs (right) showing the frequency and total number of Tfh cells from WT and *Il10R*$^{-/-}$ B cell MBM chimeric mice. (**B**) Summary graphs showing the relative titer of virus-specific IgM and IgG3 in the sera of experimental mice (**C**) Summary data showing correlation between relative antibody titers and viremia in experimental mice. (**D**) Flow histograms and summary graphs depicting the relative expression of Bcl-6, Blimp-1, and T-bet in GL-7^{+}Fas^{+} B220^{+} GC B cells from experimental mice on day 21 p.i. Summary data (mean +/- S.D. in A-C) are from 5 mice per group and were analyzed using unpaired student's t tests. Data are representative of 2 independent experiments. (N.S.= not statistically significant). Data were analyzed using two-tailed unpaired student's t tests (Supplemental Figure 6A-B,D) or linear regression (Supplemental Figure 6C). *P<0.05, ** P<0.01, *** P<0.0001, (N.S.= not significant).

Xin et al. Supplementary Figure 7

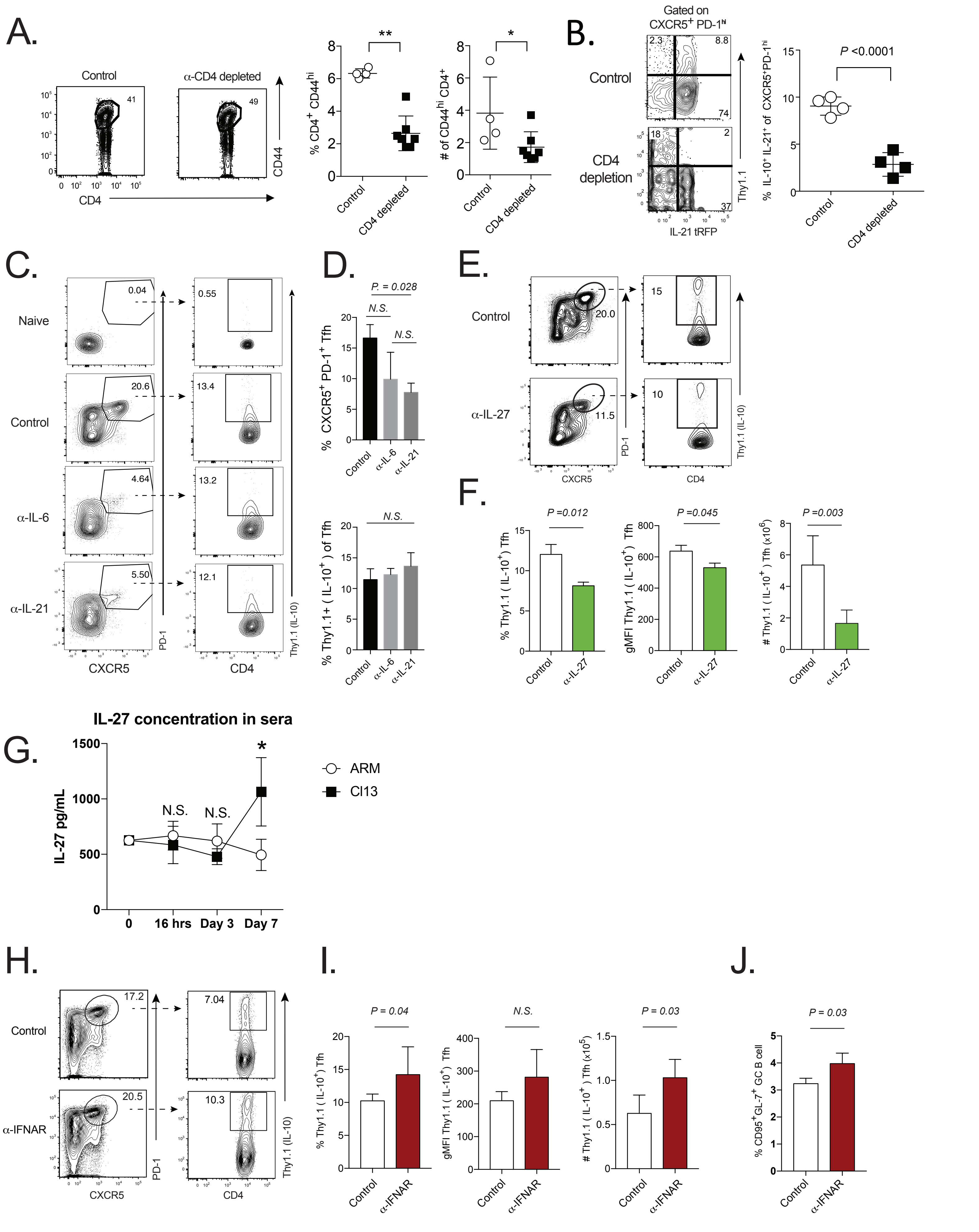

Supplementary Figure 7. Early antigenic and inflammatory signals contribute to IL-10$^+$Tfh development during LCMV Cl13 infection. Related to Figure 6.

(**A-B**) *10BiT-Il21-RFP* mice were administered either control or α-CD4-depletion antibodies and infected with LCMV Cl13 one day later. (**A**) Representative plots and summary data showing the proportion and number of CD44^{hi}CD4 T-cells on day 21 p.i. (**B**) Co-expression of IL-10 and IL-21 was examined in Tfh cells. (**C-D**) *10BiT-Il21-RFP* mice were infected with LCMV Cl13 and 500 µg of either isotype control, anti-IL-6 (Biolegend) or anti-IL-21 neutralizing antibodies were administered on days 1, 5, and 9 p.i. and CXCR5$^+$PD-1^{hi}CD44^{hi} Tfh cells were assessed for IL-10 production on day 14 p.i. Representative flow plots (C) and summary data (D) showing the proportion of Tfh cells and subsequent IL-10(Thy1.1) expression. (**E-F**) *10BiT-Il21-RFP* mice were infected with LCMV Cl13 and treated with either control or α-IL-27p28 blocking antibodies on days 3 and 9 p.i. Representative flow plots (E) and summary data (F) depicting the proportion and total number of Thy1.1$^+$(IL-10)$^+$ Tfh cells from control and α-IL-27p28-treated mice on day 14 p.i. The relative expression (gMFI) of IL-10(Thy1.1) in Tfh cells from experimental mice is also depicted (F). (**G**) WT mice were infected with LCMV Arm or Cl13. (G) Summary graph showing serum levels of IL-27 in naïve mice or experimental mice at 16 hours, 3 days or one week p.i. (**H-J**) *10BiT-Il21-RFP* mice were infected with LCMV Cl13 and treated with either control or α-IFNAR blocking antibodies on days 10, 14, and 18 p.i. (**H-I**). Representative flow plots (H) and summary data (I) depicting the proportion and number of Thy1.1(IL-10)$^+$CXCR5$^+$PD-1$^+$CD44^{hi} Tfh cells from control and α-IFNAR-treated mice on day 21 p.i. The relative expression of IL-10 (Thy1.1) in Tfh cells from experimental mice is also shown in (I). (J) Summary graph showing the proportion of CD95$^+$GL-7$^+$GC B cells. Summary data (mean +/- S.D. in A-I are from 3-5 mice/group and are representative of 2-3 independent experiments. Data were analyzed using two-tailed unpaired student's t tests (Supplemental Figure 7A-B,F-G, I-J) or one-way ANOVA (Supplemental Figure 4D). *P<0.05, ** P<0.01, *** P<0.0001, (N.S.= not significant).

Supplementary Table 1 : Antibody List

Antigen	Dilution	Clone	Fluorochrome	Source
CD4	1:200	GK1.5	Fitc	Biolegend
CD44	1:1000	IM7	APC/cy7, Percp	Biolegend
PD-1	1:200	RMP1-30	PEcy7	Biolegend
Th1.1	1:300	OX-7	Pacblue, Percp	Biolegend
CXCR5	1:100	L138D7	APC, APC/cy7	Biolegend
CXCR5	1:100	2G8	rat anti- mouse	BD Pharm
B220	1:200	RA3-6B2	APC	Biolegend
CD95 (fas)	1 in 400	15A7	PE	eBioScience
GL-7	1 in 400	GL7	Fitc	Biolegend
ICOS	1:200	C398.4A	Pacblue	Biolegend
Bcl-6	1:100	K112-91	AF647	Biolegend
tRFP	1:50		rabbit anti-mouse	Evrogen
IgD	1:50	11-26c.2a	BV421	Biolegend
Foxp3	1:100	MF-14	AF647	Biolegend
CD25	1:200	PC61	APC	Biolegend
CTLA4	1:100	UC10-4B9	PEcy7	Biolegend
GITR	1:100	DTA-1	Percp	Biolegend
T-bet	1:100	4B10	APC	Biolegend
Blimp-1	1:100	clone 5E7	AF647	Biolegend
Strepavidin	1:1000		APC	Biolegend
Donkey anti-rabbit	1:200		AF594	Life Technologies
anti-fluorescein/Oregon Green	1:200		AF488	Life Technologies
