# Supplementary material for: Single-cell RNA sequencing unveils an IL-10-producing helper subset that sustains humoral immunity during persistent infection
Source: Nat Commun. 2018 Nov 28;9:5037. doi: 10.1038/s41467-018-07492-4 (PMC6261948; doi:10.1038/s41467-018-07492-4)
Supplement: Supplementary file 2 — Reporting Summary [file 41467_2018_7492_MOESM2_ESM.pdf]

nature research

Corresponding author(s): Weiguo Cui and Ryan Zander

Reporting Summary

Nature Research wishes to improve the reproducibility of the work that we publish. This form provides structure for consistency and transparency in reporting. For further information on Nature Research policies, see Authors & Referees and the Editorial Policy Checklist.

Statistical parameters

When statistical analyses are reported, confirm that the following items are present in the relevant location (e.g. figure legend, table legend, main text, or Methods section).

n/a	Confirmed	
☐	☒	The exact sample size (*n*) for each experimental group/condition, given as a discrete number and unit of measurement
☐	☒	An indication of whether measurements were taken from distinct samples or whether the same sample was measured repeatedly
☐	☒	The statistical test(s) used AND whether they are one- or two-sided<br>*Only common tests should be described solely by name; describe more complex techniques in the Methods section.*
☐	☒	A description of all covariates tested
☐	☒	A description of any assumptions or corrections, such as tests of normality and adjustment for multiple comparisons
☐	☒	A full description of the statistics including central tendency (e.g. means) or other basic estimates (e.g. regression coefficient) AND variation (e.g. standard deviation) or associated estimates of uncertainty (e.g. confidence intervals)
☒	☐	For null hypothesis testing, the test statistic (e.g. *F*, *t*, *r*) with confidence intervals, effect sizes, degrees of freedom and *P* value noted<br>*Give P values as exact values whenever suitable.*
☒	☐	For Bayesian analysis, information on the choice of priors and Markov chain Monte Carlo settings
☒	☐	For hierarchical and complex designs, identification of the appropriate level for tests and full reporting of outcomes
☐	☒	Estimates of effect sizes (e.g. Cohen's *d*, Pearson's *r*), indicating how they were calculated
☐	☒	Clearly defined error bars<br>*State explicitly what error bars represent (e.g. SD, SE, CI)*

Our web collection on statistics for biologists may be useful.

Software and code

Policy information about availability of computer code

Data collection	All flow cytometry data were acquired on an BD LSR-II (BD Biosciences, CA). The 10X Genomics Chromium Controller was used to generate a single cell RNA-seq library.
Data analysis	All flow cytometry data were analyzed using FlowJo (Treestar, OR). Statistical tests were performed using Graphpad Prism 7. RNA-seq data were aligned using TopHat and differential gene expression was determined using the Cuffdiff function of Cufflinks. Single-cell RNA-seq data were processed using Cell Ranger (10x Genomics) and analyzed using Seurat.

For manuscripts utilizing custom algorithms or software that are central to the research but not yet described in published literature, software must be made available to editors/reviewers upon request. We strongly encourage code deposition in a community repository (e.g. GitHub). See the Nature Research guidelines for submitting code & software for further information.

Data

Policy information about availability of data

All manuscripts must include a data availability statement. This statement should provide the following information, where applicable:
- Accession codes, unique identifiers, or web links for publicly available datasets
- A list of figures that have associated raw data
- A description of any restrictions on data availability

The bulk RNA-seq and single-cell RNA-seq data have been deposited in the GEO database with the accession code GSE111027 [https://www.ncbi.nlm.nih.gov/geo/query/acc.cgi?acc=GSE111027]
A reporting summary for this Article is available as a Supplementary Information file. All other relevant raw data are available from the corresponding author upon request.

Field-specific reporting

Please select the best fit for your research. If you are not sure, read the appropriate sections before making your selection.

☒ Life sciences ☐ Behavioural & social sciences ☐ Ecological, evolutionary & environmental sciences

For a reference copy of the document with all sections, see nature.com/authors/policies/ReportingSummary-flat.pdf

Life sciences study design

All studies must disclose on these points even when the disclosure is negative.

Sample size	Samples sizes were determined based on the principle of using the minimum number of animals to provide adequate statistical power while being mindful of the recommendations from IACUC.
Data exclusions	No data were excluded
Replication	All experimental findings were reliably reproduced and all experiments are representative of at least two independent experiments with n=3-5 mice per group per experiment.
Randomization	All experimental mice were randomly allocated.
Blinding	No blinding was used.

Reporting for specific materials, systems and methods

Materials & experimental systems

n/a	Involved in the study	
☐	☒	Unique biological materials
☐	☒	Antibodies
☐	☒	Eukaryotic cell lines
☒	☐	Palaeontology
☐	☒	Animals and other organisms
☒	☐	Human research participants

Methods

n/a	Involved in the study	
☒	☐	ChIP-seq
☐	☒	Flow cytometry
☒	☐	MRI-based neuroimaging

Unique biological materials

Policy information about availability of materials

Obtaining unique materials	IL-21tRFP mice were provide by Dr. Joe Craft from Yale University. The anti-IL-27p28 blocking antibodies were provided by Dr. William R. Drobyski from the Medical College of Wisconsin.

Antibodies

Antibodies used	All flow cytometry antibodies used in this study are commercially available, and they were used only on species for which they have been validated by the vendor.<br>Anti-mouse Thy1.1 (clone 19E12 from BioXCell, Catalog # BE0214)<br>Anti-CD4 antibody (clone GK1.5 from BioXCell, Catalog #BE0003-1)<br>Anti-IFNAR antibody (clone MAR1-5A3; (BioXcell, Catalog #BE0241)<br>anti-IL-27p28 blocking antibodies (clone MM27.7B1, provide by Dr William R. Drobyski)<br>rat anti-mouse CXCR5 antibody (clone 2G8, BD, Catalog #551960)<br>APC anti-mouse CXCR5 antibody (clone L138D7, biolegend, Catalog #145506)<br>PE/Cy7 anti-mouse CD279 (PD-1) Antibody (Clone RMP1-30, biolegend,Catalog #109110)<br>PerCP anti-mouse/human CD44 Antibody (Clone IM7, biolegend, catalog # 103036)<br>Pacific Blue™ anti-rat CD90/mouse CD90.1 (Thy-1.1) Antibody (Clone OX-7,biolegend, catalog # 202522)<br>FITC anti-mouse CD4 Antibody (Clone GK1.5, biolegend, catalog # 100406)<br>Pacific Blue™ anti-human/mouse/rat CD278 (ICOS) Antibody (Clone C398.4A, biolegend, catalog # 313522)<br>APC anti-human/mouse Bcl-6 Antibody (Clone 7D1, biolegend, catalog # 358506)<br>PE/Cy7 anti-mouse/human CD45R/B220 Antibody (Clone RA3-6B2,biolegend, catalog # 103222)<br>FITC anti-mouse/human GL7 Antigen (T and B cell Activation Marker) Antibody (Clone GL7,biolegend, catalog # 144604)<br>PE anti-mouse CD95 (clone 15A7,biolegend, catalog # 12095183)<br>PE anti-mouse Foxp3 (clone MF-14 , biolegend, catalog #126403)<br>All additional antibodies used are listed in Supplementary Table 1.
Validation	All flow cytometry antibodies used in this study are commercially available, and they were used only on species for which they have been validated by the vendor.

Eukaryotic cell lines

Policy information about cell lines

Cell line source(s)	The BHK-21 cell line was purchased from American Type Culture Collection (ATCC).
Authentication	We have not authenticated the BHK-21 cell line, but this cell line was authenticated by ATCC.
Mycoplasma contamination	The BHK-21 cell line was not tested for mycoplasma contamination.
Commonly misidentified lines<br>(See ICLAC register)	BHK-21 is not on the list.

Animals and other organisms

Policy information about studies involving animals; ARRIVE guidelines recommended for reporting animal research

Laboratory animals	All mice were bred and maintained in a closed breeding facility, and mouse handling conformed to the requirements of the Institutional Animal Care and Use Guidelines of Medical College of Wisconsin. C57BL/6, Il10r-/-, Il10-/-, and Cd4–/– mice were purchased from Jackson Laboratory (Bar Harbor, ME). Il21-/- mice were kindly provided by Alan Zajac. The IL-10 and IL-21 double reporter mice were generated by crossbreeding IL-21tRFP mice with 10 BiT mice (kindly provided by Dr. Casey Weaver, University of Alabama at Birmingham, AL). For mixed bone marrow chimera experiments, recipient mice were irradiated with 6.5 and 5.5 Gy separated by 8 hours. Bone marrow from various donor mice (as depicted in Figures) were mixed at the indicated ratios, and a total of ~ 6x106 cells were transferred i.v. Mice were maintained on oral sulfamethoxazole for 2 weeks. Chimerism was assessed at 7 weeks in peripheral blood using congenic markers. Chimerism in the CD4 T cell compartment in mice reconstituted with bone marrow from Cd4-/- mice, Sh2d1a-/- mice, and either Il10-/- or WT mice was approximately 55% Sh2d1a-/- and 45% either Il10-/- or WT amongst 20 experimental mice in two independent experiments. Chimerism in the B cell compartment in mice reconstituted with bone marrow from µMT mice + bone marrow from either Il10rb-/- or WT mice was greater than 93%. Experimental MBM mice were infected with LCMV Cl13 at 8 weeks post-reconstitution.
Wild animals	No wild animals were used in this study
Field-collected samples	There were not any field-collected samples used in this study.

Flow Cytometry

Plots

Confirm that:

☒ The axis labels state the marker and fluorochrome used (e.g. CD4-FITC).

☒ The axis scales are clearly visible. Include numbers along axes only for bottom left plot of group (a 'group' is an analysis of identical markers).

☒ All plots are contour plots with outliers or pseudocolor plots.

☒ A numerical value for number of cells or percentage (with statistics) is provided.

Methodology

Sample preparation	Mouse splenocytes were subjected to red blood cell lysis, washed and subsequently stained using GP61-80 tetramer reagents (NIH) for one hour at room temperature before performing additional surface staining using fluorescently labeled antibodies. In some experiments, transcription factor staining was performed using True Nuclear transcription factor buffer set from Biolegend.
Instrument	BD LSR-II Flow cytometer
Software	FlowJo Version 9
Cell population abundance	The purity of cells sorted for in vitro co-cultures was greater than 90% as tested by running post-sort samples on FACS machine.
Gating strategy	For all flow cytometry experiments, forward and side scatter measurements were used to pre-gate on the lymphocyte population, before proceeding to gate on singlets using side scatter height and width measurements. Cells that stained positive for CD4 were gated on for additional downstream analyses as depicted/indicated in figures.

☒ Tick this box to confirm that a figure exemplifying the gating strategy is provided in the Supplementary Information.
